# The gut microbiota protein BOC1 exhibits immune checkpoint inhibitor-like activity by inhibiting myeloid-derived suppressor cell differentiation

**DOI:** 10.3389/fimmu.2025.1607543

**Published:** 2025-08-19

**Authors:** Laureen Bardouillet, Maria Lucia Orsini Delgado, Caroline Matondo, Francesco Strozzi, Valentine Thomas, Laurent Chene, Antonietta Cultrone

**Affiliations:** Drug Discovery Department, Enterome, Paris, France

**Keywords:** gut microbiota, immune check-point inhibitor, CD200R1, MDSC, cancer immunotherapy

## Abstract

**Background:**

Advancing research in oncology highlights the inverse correlation between antibiotic treatment and the positive outcomes of immune checkpoint inhibitor (ICI) administration, confirming once more the importance of microbiota and microbiota-derived compounds as complementary tools for treating cancer. Among the immune checkpoints, the CD200 cell surface glycoprotein has gained attention for its role in promoting self-tolerance and potentially facilitating tumor growth through interaction with the CD200R1 receptor.

**Methods:**

We developed a robust AlphaLISA-based screening to identify human gut microbiota-derived proteins that may interact with CD200R1 and screened a library of 10,966 gut bacterial proteins. The antitumor activity of BOC1 was investigated *in vitro* by cytokine analysis, mixed lymphocyte reactions, and myeloid-derived suppressor cell (MDSC)–T-cell suppression assay. AlphaFold modeling was used to predict potential interaction points between BOC1 and CD200R1.

**Results:**

We successfully identified BOC1, a protein from the *Bacteroides* genus, showing better affinity than the natural ligand, CD200, toward the CD200R1 receptor. BOC1 induces cytokine secretion by monocyte-derived dendritic cells (MoDCs) and enhances CD8^+^/CD4^+^ T-cell populations and IFNγ production, highlighting its potent immunostimulatory properties. BOC1 also negatively impacts the differentiation of MDSCs, maintaining an immature monocytic profile (high CD14 and HLA-DR expression) and restoring T-cell proliferation even at low (10 nM) concentration. Mutation of amino acids within the N-terminal region of BOC1 reduces binding to CD200R1, supporting the importance of this region for a possible interaction with CD200R1.

**Conclusion:**

The immunostimulatory properties of BOC1 observed *in vitro* are compatible with an ICI-like behavior of this bacterial protein. Given that neither the CD200 protein nor the anti-CD200 antibody is able to compete with BOC1 for binding to CD200R1, and as supported by AlphaFold modeling predictions, CD200 and BOC1 might target different regions of CD200R1.

## 1 Introduction

The tumor microenvironment (TME) is a densely vascularized extracellular matrix (ECM) comprising cancer and healthy cells, where different cell types display either immunosuppressive or antitumoral function (1, 2). Under normal conditions, immune cells, mainly cytotoxic T cells, recognize tumor-specific antigens presented by MHC class I molecules and kill cancer cells. However, in the TME, malignant cells have “learned” to evade the immune response, reprogramming the surrounding cells to secrete anti-inflammatory factors that promote a tolerogenic environment favorable to cancer progression (3–5). Suppression of CD8^+^ T-cell responses in the TME often occurs through direct interactions between T cells and cancer cells or other neighboring cells. This process frequently involves immune checkpoint pathways, with well-documented examples including PD-1/PD-L1 and CTLA-4/B7-1/B7–2 interactions, among others (6–8). Antibodies blocking these interactions have been developed and validated in clinical trials to reactivate T-cell immune surveillance and ultimately treat cancer (9–11). Even if immune checkpoint inhibitor (ICI) combinations have been associated with adverse effects, sometimes leading to treatment cessation (12), their generally promising outcomes push research to seek new molecules, other than antibodies, that might improve patient response to immunotherapy.

We focused our research on the CD200/CD200R1 axis, recently pointed out as potential targets in some forms of cancer (13, 14). CD200R1, a receptor belonging to the immunoglobulin superfamily, is mainly expressed on dendritic cells (DCs), myeloid-derived suppressor cells (MDSCs), and macrophages, but also B and T lymphocytes. A positive interaction between CD200R1 and CD200 (also named OX-2) causes a strong immune-suppressive response and was initially evaluated as a natural treatment regulating transplant rejection (15) and other autoimmune disorders (16–18). Furthermore, CD200/CD200R1 interaction in the TME fosters MDSC and regulatory T-cell (Treg) differentiation, which suppresses antitumor immunity (19, 20). High levels of CD200 have been found to be expressed on various types of cancer cells, including some B-cell malignancies such as hairy cell leukemia, chronic lymphocytic leukemia (CLL), and multiple myeloma (MM), but also malignant melanoma, colon carcinoma, glioblastoma (GBM), and pancreatic ductal adenocarcinoma (PDAC), and were generally correlated with poor patient prognosis (21–24). In addition, a soluble form of CD200 (sCD200), released following CD200 ectodomain shedding by matrix metalloproteinase-1 (MMP1) and ADAM19 metallopeptidase, is able to engage CD200R1, can be quantified in the serum, and has been correlated with worse tumor prognosis in CLL (25) and GBM patients (20). Furthermore, in both GBM and PDAC, increased levels of CD200 were associated with MDSC population expansion (20, 23). MDSCs are described to downregulate the antitumor immune activity, and at least in pancreatic cancer, the number of MDSCs correlates with a poor overall survival. Thus, breaking CD200/CD200R1 interaction might reinvigorate T-cell responses, boosting the antitumor activity. Two different approaches, targeting the CD200/CD200R1 interaction, are currently under investigation: using monoclonal antibodies or using inhibitory peptides. Samalizumab is the first recombinant humanized monoclonal antibody targeting CD200 that has been used in a cohort of patients with advanced CLL and MM (26). Moreover, results from a first-in-human phase 1/2a study of 23ME-00610, an antibody targeting CD200R1 in solid malignancies, have just been published (27). While encouraging, even if not long-lasting, results were observed (decreased tumor burden observed in treated CLL patients), and mild to moderate adverse effects were also monitored. First attempts to break the CD200/CD200R1 interaction with small peptides mimicking regions of CD200 ligands date from 2005 by Chen et al. (28). Starting from the demonstration that CD200R1 is closely related to CD200, the authors demonstrated that small CD200-derived peptides blocked the interaction between CD200 and CD200R1 *in vitro* and the suppression of graft rejection *in vivo* in a skin graft rejection model (28). Since then, other teams successfully confirmed the potential of this approach (29).

Increasing literature emphasizes the role of gut microbiota in enhancing the outcome of immunotherapy while reducing the adverse events linked to these treatments. Encouraging results from Vétizou et al. (30) indicated a positive correlation between anti-CTLA-4 blockade and the presence of *Bacteroides thetaiotaomicron* or *Bacteroides fragilis* in a sarcoma mouse model. Similarly, the study of Sivan and coworkers showed that melanoma-induced mice exhibited improved response to a combined treatment including anti-PD-L1 administration together with a transfer of fecal material enriched in *Bifidobacterium* species (31). In the generation of personalized medicine, microbiota composition can be modified through pre-/probiotic and antibiotic administration or fecal microbiota transplantation (FMT). Alternatively, the administration of bacteria-derived compounds is evaluated to further boost anticancer therapies (32, 33). With the aim of identifying bacterial proteins that interact with CD200R1 and potentially interfere with the natural CD200/CD200R1 binding, we developed an AlphaLISA™, as a robust tool for high-throughput screening (HTS). Our screening revealed BOC1, a bacterial protein derived from the human gut microbiota, which effectively interacts with CD200R1. BOC1 was shown to modulate cytokine secretion by human monocyte-derived dendritic cells (MoDCs) and to function as an immunostimulatory element, increasing CD8^+^ and CD4^+^ cell populations and IFNγ secretion. This immunostimulatory property is in line with the capacity of BOC1 to inhibit monocyte differentiation into MDSCs and restore T-cell proliferation. AlphaFold prediction suggests that BOC1 and CD200 may, respectively, interact within close but not overlapping regions of CD200R1, and by using BOC1 mutants, we could identify some residues presumably involved in the BOC1/CD200R1 interaction. Altogether, these results represent a starting point for future evaluation *in vivo* of BOC1 as a potential ICI, specifically targeting the CD200/CD200R1 axis.

## 2 Materials and methods

### 2.1 Cell-free synthesis and screening

A protein library was generated using the PUREsystem (Protein synthesis Using Recombinant Elements) cell-free technology (PUREfrex.2, GeneFrontier, Kashiwa, Japan) in which a mix containing the synthesis machinery of *Escherichia coli* (purified factors involved in transcription, translation, and energy regeneration) is incubated with the target DNA. All the genes of the cell-free library were synthesized and cloned into a pIVEX 2.4d vector (Twist Bioscience, South San Francisco, CA, USA) with an N-terminal His-tag. The library was generated in 384-well plates in a volume of 20 µL per well. A total of 10 ng of DNA was used per 20 µL reaction, and additional components such as disulfide bond isomerase (DsbC) and protein chaperones (DnaK and GroE) were added to improve the synthesis. Plates were incubated for 4 h at 37°C under agitation and then were removed from the incubator, and 30 µL of PBS was added to each well with a multidrop (these plates were named “mother plates”). Eight “stimulation plates” (used for the screening) were generated from the mother plates by diluting 5 µL of each synthesis with 45 µL of PBS. Both the mother and stimulation plates were stored at −80°C.

The screening was run in duplicate on 384-well plates in a volume of 20 µL per well. All the reagents were prepared in ImmunoAssay buffer (Revvity, Waltham, MA, USA). Five microliters (0.3 nM) of biotinylated CD200R1 was added per well using a multidrop (Thermo Fish Scientific Inc., Waltham, MA, USA) and then 5 µL of proteins from the “stimulation plate” were dispensed with a Hamilton Star robot. Assay plates were incubated at room temperature for 1 h. A total of 10 µg/mL of anti-6His Acceptor Beads (Revvity, Waltham, MA, USA) and 10 µg/mL of streptavidin Donor beads (Revvity, Waltham, MA, USA) were successively added with an automated dispenser under limited light conditions, and the plates were incubated again at room temperature in the dark for 1 h. The AlphaLISA signal was measured using an EnVision reader (Revvity, Waltham, MA, USA). Screening results were analyzed with Spotfire (TIBCO; Palo Alto, CA, USA). We considered positive those proteins showing a signal 3-fold higher than the mean of the plates.

### 2.2 Protein purification

Proteins meant to be purified were synthesized (cell-free) at a higher scale in Eppendorf tubes using 800 to 1,200 µL of PUREfrex.2 (GeneFrontier, Kashiwa, Japan) mix (see details in subsection 2.1) and 400 to 600 ng of DNA (depending on yield requirement) and purified using the HisPur Ni-NTA superflow agarose beads (Thermo Fish Scientific Inc., Waltham, MA, USA) according to the provider’s protocol. Imidazole, used for protein elution, was removed by successive centrifugations (10 min × 14,000*g*) with 10-kDa cutoff concentrators (Amicon^®^). Protein expression and purity were assessed by SDS-PAGE and Coomassie Blue staining. Purified proteins were also visualized by Western blot using a 6His-tag monoclonal antibody (HIS.H8, Thermo Fish Scientific Inc., Waltham, MA, USA) and a goat anti-mouse IgG secondary antibody (H+L) (Thermo Fish Scientific Inc., Waltham, MA, USA). Proteins were quantified by the Bradford method using a BSA standard curve (0.1–1.2 mg/mL). Protein aliquots were stored at −80°C (in PBS 1× + 5% glycerol).

### 2.3 Synthesis of small fragments

Seven small fragments (F1 to F7), 50 amino acids long, covering the complete BOC1 sequence were synthesized (>98% purity) by the solid-phase peptide synthesis (SPPS) methodology (Smart Bioscience, Saint-Égrève, France). Both stock (100 µM) and working solutions were made in ultrapure H_2_O. Peptide F7, showing poor solubility, was excluded from the analysis.

### 2.4 AlphaLISA competition assay

A competition assay was designed firstly to verify that the untagged CD200 (CD200-Fc) could interfere with the CD200-His/CD200R1-(biot) complex. Secondly, we investigated the capacity of short BOC1 peptides to displace the CD200-His/CD200R1-(biot) interaction. For this, 0.3 nM of CD200R1-(biot) and 3 nM of CD200-His were incubated with a dose response of CD200-Fc (0.3 to 300 nM) or each short-BOC1 peptide (0.01 to 1 µM) for 1 h at room temperature (RT). At the end of the incubation time, 10 µg/mL of anti-6His Acceptor Beads (Revvity, Waltham, MA, USA) and 10 µg/mL of streptavidin Donor beads (Revvity, Waltham, MA, USA) were added to each well, and the plates were incubated again at RT in the dark for 1 h. The AlphaLISA signal was measured using an EnVision reader (Revvity, Waltham, MA, USA). CD200-Fc or short-BOC1 peptide capacity to displace the CD200-His/CD200R1-(biot) complex was expressed either as AlphaLISA counts or as dissociation percentage with respect to the maximum signal obtained from CD200-His/CD200R1-(biot) interaction.

### 2.5 Human primary cell isolation

Blood samples from healthy donors were purchased from Etablissement Français du Sang (EFS, Pontoise, France). Peripheral blood mononuclear cells (PBMCs) were isolated from buffy coat by density gradient centrifugation. Blood from each buffy coat was diluted with 80 mL of Dulbecco’s phosphate-buffered saline (DPBS) without calcium and magnesium (Sigma-Aldrich^®^, Merck KGaA, Darmstadt, Germany) supplemented with 2% FBS (Sigma-Aldrich^®^, Merck KGaA, Darmstadt, Germany) and 1 nM of EDTA (Sigma-Aldrich^®^, Merck KGaA, Darmstadt, Germany). Then, 30 mL of diluted blood was carefully layered over 15 mL of Ficoll^®^-Paque PLUS (Cytiva, Marlborough, MA, USA) in four SepMate™ 50 mL tubes (STEMCELL Technologies, Saint-Égrève, France) and centrifuged at 1,200×*g* for 20 minutes with reduced acceleration and deceleration. The PBMC layers were then transferred to a 50-mL Falcon tube, washed three times with 45 mL of DPBS 1×, and recovered by centrifugation at 300×*g* for 10 min. After the last centrifugation, PBMCs were treated for 10 min at room temperature with 1× Red Blood Cell Lysis solution (Miltenyi Biotec, Bergisch Gladbach, Germany) to eliminate residual erythrocytes. Finally, PBMCs were counted with a LUNA-FL™ fluorescence cell counter using Acridine Orange/Propidium Iodide Stain for viability assessment (Logos Biosystems by Aligned Genetics, Inc., Villeneuve d’Ascq, France). CD14^+^ and CD3^+^ cells were purified by positive selection using CD14 MicroBeads human (Miltenyi Biotec, Bergisch Gladbach, Germany) and CD3 MicroBeads human (Miltenyi Biotec, Bergisch Gladbach, Germany) magnetic isolation beads, following the manufacturer’s instructions. Purity was determined by flow cytometry.

### 2.6 Cell differentiation

#### 2.6.1 Cell culture media

Complete Iscove’s modified Dulbecco’s medium (IMDM) was prepared by supplementing 1× IMDM (Gibco™ Thermo Fisher Scientific Inc., Waltham, MA, USA) with 10% FBS, 1% penicillin–streptomycin (Sigma-Aldrich^®^, Merck KGaA, Darmstadt, Germany), and 1% GlutaMAX (Gibco™ Thermo Fisher Scientific Inc., Waltham, MA, USA). Complete ImmunoCult medium was prepared by supplementing ImmunoCult™-XF T Cell Expansion Medium (STEMCELL Technologies, Saint-Égrève, France) with 1% penicillin–streptomycin (Sigma-Aldrich^®^, Merck KGaA, Darmstadt, Germany).

#### 2.6.2 Monocyte-derived dendritic cell differentiation

Human primary CD14^+^ cells, isolated as described above, were differentiated into MoDCs by cultivating 3.5 × 10^7^ cells in 20 mL of complete IMDM in the presence of 20 ng/mL of recombinant human interleukin 4 (rhIL-4) premium grade and 20 ng/mL of recombinant human granulocyte macrophage colony-stimulating factor (rhGM-CSF) premium grade (Miltenyi Biotec, Bergisch Gladbach, Germany). Cells were incubated for 7 days at 37°C, 5% CO_2_; recovered by soft flushing; and used for functional assays.

#### 2.6.3 Myeloid-derived suppressor cell differentiation

To induce myeloid-derived suppressor cell (MDSC) differentiation, 10^6^ monocytes were distributed per well in a 24-well culture-treated plate (Falcon^®^, Corning Inc., Corning, NY, USA) in complete IMDM in the presence of 10 ng/mL of recombinant human interleukin 6 (rhIL-6) premium grade and 10 ng/mL of rhGM-CSF (Miltenyi Biotec, Bergisch Gladbach, Germany). The medium was refreshed on days 2 and 5, and cells were incubated for a total of 7 days at 37°C, 5% CO_2_. When indicated, BOC1 or P352 proteins were added at the indicated final concentrations. Subsequently, cells were recovered using enzyme-free PBS-based cell dissociation buffer (Gibco™ Thermo Fisher Scientific Inc., Waltham, MA, USA) following the supplier’s instructions. The cell phenotype was analyzed by flow cytometry and used in functional assays.

#### 2.6.4 T-cell activation

For T-cell activation assays, 10^5^ cells were distributed per well in a 96-well clear round bottom untreated microplate (Corning Inc., Corning, NY, USA) in 200 µL of complete ImmunoCult medium in the presence of ImmunoCult™ human CD3/CD28 T Cell Activator (STEMCELL Technologies, Saint-Égrève, France) according to the supplier’s recommended dilution (25 µL/mL) and 100 IU/mL of recombinant human interleukin 2 (rhIL-2) premium grade (Miltenyi Biotec, Bergisch Gladbach, Germany). Cells were incubated for 4 days at 37°C, 5% CO_2_, and recovered for CD200/CD200R1 expression assessment and protein binding assays by flow cytometry.

### 2.7 *In vitro* functional assays

#### 2.7.1 Cytokine profiling with LEGENDplex multiplex technology

MoDCs were plated in 96-well plates at a density of 45,000 cells per well using complete IMDM culture medium. Cells were stimulated with BOC1 or P352 proteins at 0.5 µM in 100 µL per well for 24 h. Following incubation, the supernatants were collected and cryopreserved at –20°C prior to cytokine quantification.

Cytokine quantification was performed using the LEGENDplex™ bead-based immunoassay (human inflammation Panel 1, BioLegend, San Diego, CA, USA) following the manufacturer’s specifications. Briefly, the LEGENDplex™ multiplex flow cytometry assay utilizes antibody-conjugated fluorescent beads with distinct spectral signatures, enabling simultaneous detection of multiple analytes through a capture antibody-biotinylated detection sandwich system, followed by PE-streptavidin signal amplification. Samples were acquired on a MACSQuant^®^ Analyzer flow cytometer (Miltenyi Biotec, Bergisch Gladbach, Germany) and analyzed using GraphPad Prism software, with cytokine concentrations determined through 5-parameter logistic (5PL) regression modeling of standard curves.

#### 2.7.2 IFNγ and TNFα measurement with AlphaLISA technology

IFNγ and TNFα were quantified through the AlphaLISA technology (Revvity, Waltham, MA, USA). The provider’s protocol was adapted to be used with a robotic station (Hamilton, Reno, NV, USA) and optimized to reduce reagent consumption. Five microliters of a mix containing anti-IFNγ or anti-TNFα biotinylated antibody (0.5 nM final) and anti-IFNγ or anti-TNFα Acceptor Beads (5 µg/mL final) was added to a 384-well plate, then 5 µL of the supernatant was added to each well. After 1 h at RT, 5 µL of Streptavidin Donor Beads (20 µg/mL final) were added, and the plate was incubated again in the dark for 30 min at RT. The AlphaLISA signal was measured using an EnVision reader (Revvity, Waltham, MA, USA). A standard curve was used for concentration calculation.

#### 2.7.3 T-cell CFSE labeling

Isolated T cells were labeled with the cell proliferation tracer carboxyfluorescein succinimidyl ester (CFSE) using the CellTrace™ CFSE Cell Proliferation Kit (Thermo Fisher Scientific Inc., Waltham, MA, USA.) following the supplier’s instructions with some modifications. Briefly, CellTrace™ CFSE was resuspended using 18 µL of DMSO to obtain a 5 mM solution, then diluted in PBS 19× at 37°C to obtain a 4 µM concentration staining solution. A 10^7^-cell/mL suspension was prepared in warm PBS, and 1 volume of staining solution was added to 1 volume of cell suspension (CFSE final concentration 2 µM). Cells were incubated for 20 min at 37°C, and any unbound dye was quenched by adding the same volume of prewarmed FBS. After 5 min of incubation at 37°C, cells were centrifuged for 5 min at 300×*g*, and the cell pellet was resuspended in prewarmed complete ImmunoCult medium.

#### 2.7.4 T-cell proliferation assay (mixed lymphocyte reaction)

MoDCs differentiated as indicated above were cocultured with allogenic CFSE-labeled T cells in a 1:10 ratio (15,000 MoDCs:150,000 T cells) in a 96-well clear round-bottom untreated microplate (Corning Inc., Corning, NY, USA). When indicated, BOC1 or P352 proteins were added at 0.5 and 1 µM final concentrations. As controls, T cells were incubated in the absence of MoDCs and in the presence (proliferation control) or absence (non-proliferation control) of ImmunoCult™ human CD3/CD28 T Cell Activator and rhIL-2 at the concentrations indicated above (“T-cell activation” section). Cells were incubated for 5 days at 37°C, 5% CO_2_, and the supernatant was recovered and stored at −20°C until cytokine measurement. Proliferation was assessed by flow cytometry based on CFSE staining median fluorescent intensity (MFI) on CD4^+^ and CD8^+^ T-cell subpopulations.

#### 2.7.5 T-cell suppression assay

Monocytes, cultured in MDSC differentiation conditions in the presence or absence of BOC1 or P352, were recovered and cocultured with allogenic CFSE-labeled T cells in a 1:4 ratio (50,000 live differentiated monocytes:200,000 T cells) in a 96-well clear round-bottom untreated microplate (Corning Inc., Corning, NY, USA.) and 100 µL of complete ImmunoCult medium per well. As controls, T cells were incubated in the absence of MDSCs and in the presence (proliferation control) or absence (non-proliferation control) of ImmunoCult™ human CD3/CD28 T Cell Activator and rhIL-2 at the concentrations indicated above (“T-cell activation” section). Cells were incubated for 5 days at 37°C, 5% CO_2_, and the supernatant was recovered and stored at –20°C until cytokine measurement. Proliferation was assessed by flow cytometry based on CFSE MFI on CD4^+^ and CD8^+^ T-cell subpopulations.

### 2.8 Antibodies and flow cytometry

The following reagents were used on the flow cytometry assays: MACS buffer (autoMACS^®^ Rinsing Solution with MACS^®^ BSA Stock Solution, 0.05% final BSA concentration), human FcR blocking reagent, PerCP-vio700 anti-CD3, vioGreen anti-CD4, APC-vio770 anti-CD8, APC-vio770 anti-CD11b, PE-vio770 anti-CD14, FITC anti-CD33, vioBright B515 anti-CD200, PE-vio615 anti-CD200R, and PE anti-HLA-DR (all from Miltenyi Biotec, Bergisch Gladbach, Germany); PE anti-His-tag (BioLegend, San Diego, CA, USA); and viability dye 4′,6-diamidino-2-phénylindole dihydrochloride, 2-(4-amidinophényl)-6-indolecarbamidine (DAPI, from Sigma-Aldrich^®^, Merck KGaA, Darmstadt, Germany). For the different assays, 10^5^ cells were distributed per well in a 96-well clear round-bottom untreated microplate (Corning Inc., Corning, NY, USA). After washing with MACS buffer, cells were resuspended in 50 µL of MACS buffer containing the antibody mix of interest. After 20 min of incubation at 4°C, cells were washed and 70 µL of 1× DAPI solution was added. Cell viability, different population percentages, and median fluorescent intensity of the cell markers of interest were determined by flow cytometry (MACSQuant^®^ X Flow Cytometer from Miltenyi Biotec, Bergisch Gladbach, Germany). Analysis was performed using FlowJo software (FlowJo™ 10.8.1, BD Life Sciences, Ashland, OR, USA).

#### 2.8.1 Purity of CD3 and CD14 isolated cells

Cell purity of magnetically isolated T cells or monocytes was assessed by flow cytometry using fluorescently labeled anti-CD3 or anti-CD14 antibodies, respectively, diluted 1/100 in MACS buffer. A comparison of the positive population percentage on unsorted PBMC and sorted cells was done. Purity was considered acceptable when the positive population percentage was ≥90%.

#### 2.8.2 Myeloid-derived suppressor cell phenotype

After differentiation, the MDSC phenotype was assessed by flow cytometry. Cells were recovered and counted, and 10^5^ cells were distributed per well in a 96-well plate. A solution containing fluorescently labeled anti-CD14, anti-CD11b, anti-CD33, and anti-HLA-DR antibodies, all diluted 1/100, was prepared, and 50 µL was distributed per well. After 20 min of incubation at 4°C, cells were washed and 70 µL of 1× DAPI solution was added. Cell viability, different population percentages, and MFI of the cell markers of interest were determined by flow cytometry.

#### 2.8.3 Functional assays

After T-cell proliferation/suppression assays, CFSE-labeled T-cell proliferation was evaluated by flow cytometry. Cells were stained using fluorescently labeled anti-CD4 and anti-CD8 antibodies to identify the different T-cell subpopulations. After 20 min of incubation at 4°C, cells were washed and 70 µL of 1× DAPI solution was added. Cell viability and CFSE MFI of the CD4**^+^** or CD8**^+^** subpopulations were determined by flow cytometry.

#### 2.8.4 CD200R1 expression

To compare the level of expression of CD200 and CD200R1 on monocytes, MoDCs, MDSCs, and unstimulated or anti-CD3/CD28-stimulated CD4^+^ and CD8^+^ T cells, 10^5^ cells were distributed per well in a 96-well plate. Cells were stained using fluorescently labeled anti-CD200 and anti-CD200R1 antibodies. After 20 min of incubation at 4°C, the cells were washed and 70 µL of 1× DAPI solution was added. Cell viability and CD200 or CD200R MFI were determined by flow cytometry.

#### 2.8.5 Protein binding assay

The binding capacity of BOC1 or the negative control P352 to monocytes, MoDCs, and MDSCs was evaluated by flow cytometry. To this end, 10^5^ cells were distributed per well; after washing with MACS buffer, cells were resuspended in 50 µL of MACS buffer containing a range of concentrations (0.5 to 0.125 µM) of His-tagged BOC1 or P352. After 60 min of incubation at 4°C, cells were washed and 50 µL of MACS buffer containing 1 µL of PE anti-His-tag antibody was added. After 20 min of incubation at 4°C, cells were washed, 70 µL of 1× DAPI solution was added and PE fluorescent intensity on live cells (DAPI^−^) was determined by flow cytometry.

### 2.9 AlphaFold structure and protein–protein interaction prediction

For BOC1 structure prediction and CD200/CD200R1 and BOC1/CD200R1 interaction prediction, the AlphaFold Server (powered by AlphaFold 3) (34) and UCSF ChimeraX 1.8 (rbvi.ucsf.edu/chimerax/, Resource for Biocomputing, Visualization, and Informatics at the University of California, San Francisco) were used. The primary amino acid sequence of the proteins of interest was pasted on the AlphaFold Server. The best folding prediction image for BOC1 was generated using UCSF ChimeraX with the AlphaFold Server, which selected the top model from the five highest-scoring models. The local distance difference test (pLDDT), the predicted template modeling (pTM) score, the predicted aligned error (PAE), and the sequence coverage were obtained from the AlphaFold Server. For the generation of protein–protein interaction predictions, images were generated with UCSF ChimeraX using the AlphaFold Server interaction prediction models. The list of amino acid interactions was obtained by structure analysis of protein–protein intramodel contacts, using ChimeraX, using a center-to-center atom distance ≤4.0 Å.

### 2.10 Statistical analysis

All assays were performed at least twice unless otherwise specified. Graphs and statistical analyses were generated using GraphPad PRISM^®^ v.10.0.0 for Windows (GraphPad Software Inc., San Diego, CA). For AlphaLISA curves, a non-linear regression was applied. The statistical analysis of MDSCs, MoDCs, and mixed lymphocyte reaction (MLR) experiments was carried out using a paired *t*-test.

## 3 Results

### 3.1 Library screening and BOC1 identification

BOC1 has been identified following the screening of a library of 10,966 proteins derived from the human gut microbiota. The library was obtained starting from the protein set of the Integrated Gene Catalog (IGC, 10Mio proteins) (35). All the sequences within the length range of 50 to 350 amino acids and originating from complete genes were retained, for a total of 3,860,003 sequences. Predicted proteins in these catalogs were first screened *in silico* for the presence of a signal peptide (for the detection of secreted proteins) using hidden Markov models or neural network-based software such as Phobius or SignalP (36, 37). Indeed, proteins that are secreted by the bacteria can interact with the host cells and the immune system. The sequences showing a positive signal peptide were then processed to calculate the number of cysteines per sequence. All the proteins were then annotated using InterProScan (v.5.30-69) to identify transmembrane domains, as well as domains and motifs from the Conserved Domain Database (CDD) and the PROSITE and CATH databases. The software MMSeqs2 was also used to perform a rapid profile search with the protein sequences against the PFAM (v.31) and HAMAP (v.2018_08) databases. All the annotation information, along with the nucleotide and protein sequences and the signal peptide prediction, was compiled into a database for further data inspection and extraction. Additional sets of filters (protein length, presence of relevant functional domains, cysteine number) were also applied. The proteins were finally clustered at 75% to avoid any redundancy in the sequences. The library was generated using a cell-free synthesis process to minimize the presence of endotoxins that could interfere with the biological tests.

To identify proteins able to bind to CD200R1 that could subsequently interfere with CD200/CD200R1 signaling, a biochemical approach based on protein–protein interaction (PPI) and AlphaLISA™ technology (a bead-based technology derived from the amplified luminescent proximity homogeneous assay) (38) was used. In the assay, one of the two PPI partners is biotinylated and bound to streptavidin-coupled beads; the second partner is histidine (His)-tagged and bound to anti-6His antibody-coupled beads. In the event of an interaction between the two proteins, an AlphaLISA signal (615 nm) is generated following streptavidin-coupled bead excitation at 680 nm (**Supplementary Figure 1A**). This setting does not allow the screening of the library in a competitive way by looking at the inhibition of CD200/CD200R1 interaction (**Supplementary Figure 1B**) since the proteins of the library are tagged with a histidine tail. We addressed this issue by looking for positive interactions of potential hits (identified by chemiluminescent emission at 615 nm) with a biotinylated CD200R1 (**Supplementary Figure 1C**). Before screening a full set of 10,966 proteins, we performed a robust setup through the cross-titration of both CD200 and CD200R1 proteins (from 0.3 to 100 nM), the identification of the optimal bead concentration, and the evaluation of the impact of the matrix used for the cell-free synthesis on AlphaLISA signal, therefore allowing the full validation of the screening protocol (**Supplementary Figures 2A–D**; **Supplementary Information 1**).

**Figure 1:**
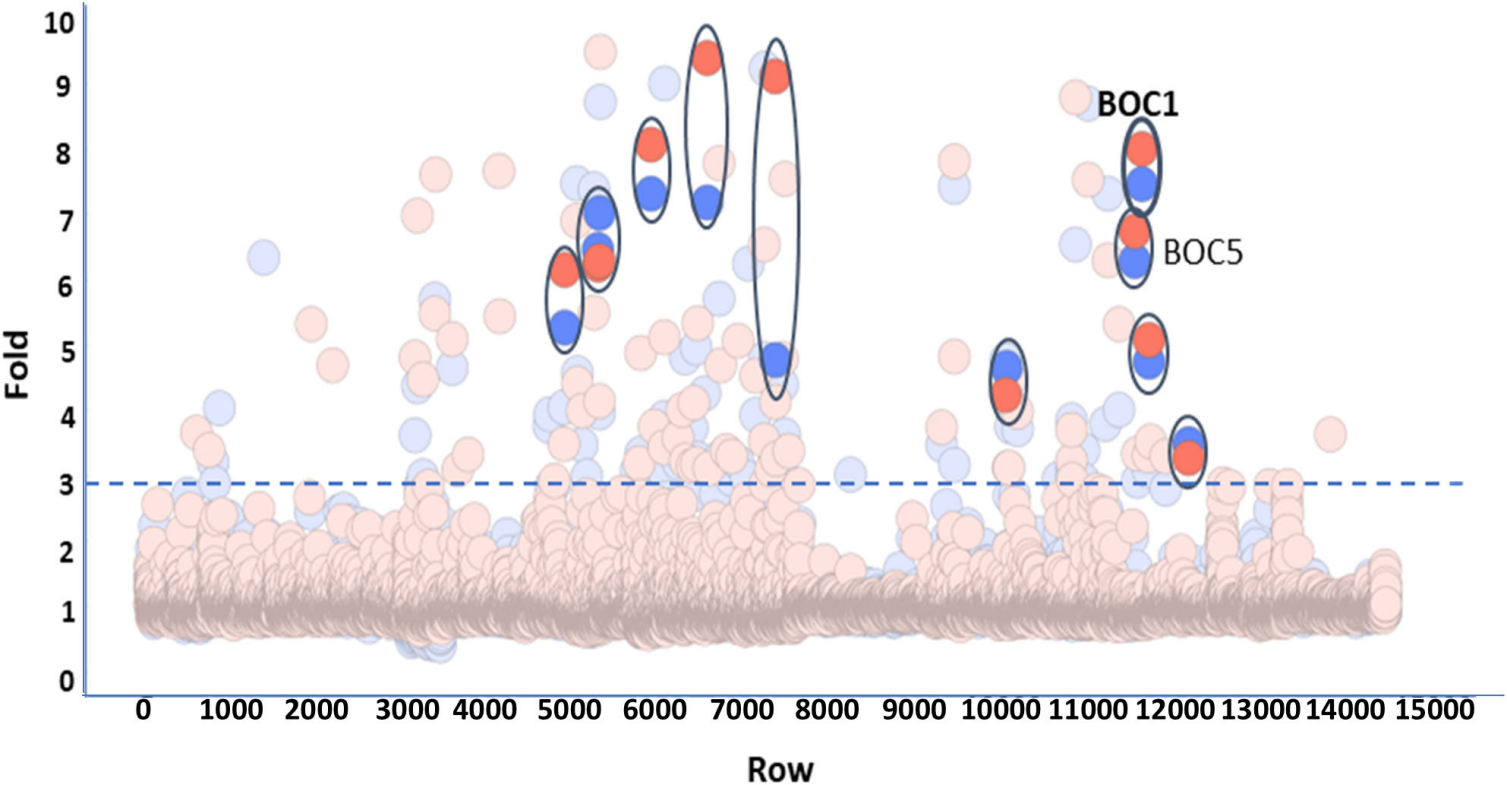
Screening of 10,966 bacterial proteins derived from the human gut microbiota. Proteins produced by cell-free technology were all tested at the same dilution (1/25). Each plate of the library was screened in duplicate. Data are expressed as fold vs. the mean of the plate. The threshold allowing the selection of positive proteins was fixed at 3. Positive proteins, confirmed by the test of a second batch, are highlighted and encircled (BOC1 and BOC5 are indicated).

Cell-free produced proteins were quantified by Homogeneous Time-Resolved Fluorescence (HTRF-6His using a GFP-His calibration curve) (**Supplementary Figure 3**). The estimated concentration was quite variable depending on the intrinsic specificity of each protein, and for technical ease, proteins were all screened at the same dilution (1/25). We considered as positive those proteins showing a signal 3-fold higher than the mean of the plates. Of the 98 proteins back positive from the primary screening, only 11 proteins were confirmed using a new synthesis batch (**Figure 1**). Among these CD200R1-binding candidates, we selected two proteins, BOC1 and BOC5, showing similar activity (6–7-fold) and sharing highly similar sequences (67% identity), indirectly confirming their effectiveness as CD200R1 binder. Among the two proteins, BOC1 was finally chosen for deeper characterization because of the higher production yield necessary to run all the characterization studies described herein.

### 3.2 Sequence analysis

Mature BOC1 is a protein of 201 amino acids from the *Bacteroides* genus, showing, respectively, 99.5% and 67% identity (Blastp analysis) with the DUF4627 domain-containing proteins of *Bacteroides gallinaceum* (strain 109) and *Phocaeicola plebeius*. Corresponding Blastp analysis of the homologous protein BOC5 (204 residues) indicates 99.5% identity with the DUF4627 domain-containing proteins of *P. plebeius*. Proteins homologous to BOC1 are ranged in two main clusters of the phylogenetic tree (**Supplementary Figure 4**), which, considering the low sequence similarity (≤30% those from the fimbrillin family), probably feature separate functions. Conversely, BOC1 is ranged in a small clade close to a large cluster of homologous proteins from *P. plebeius* (including BOC5); these proteins probably share similar functions. While *B. gallinaceum* is a species commonly detected in chickens but not in humans (39), *P. plebeius* (formerly known as *Bacteroides plebeius*) has been isolated from the human microbiota, prevalently in people of Japanese origin (40), and it is known for the ability to digest seaweed-derived sulfated polysaccharides.

### 3.3 BOC1 binding to different cell types

To follow up on the hypothesis of BOC1 as a CD200R1 ligand, we analyzed the binding of this protein to human cells. CD200R1 is described to be strongly expressed on myeloid cells, especially on MoDCs (41). CD200R1 and CD200 expressions were evaluated on different cell types (monocytes, MoDCs, MDSCs, resting and anti-CD3/CD28-stimulated CD4^+^ and CD8^+^ T cells) following incubation with an anti-CD200R1-APC-labeled or an anti-CD200-FITC-labeled antibodies. In general, a higher expression of CD200R1 compared to that of CD200 was observed in all cell types, with the highest expression on T cells and MoDCs (**Table 1**; **Supplementary Figure 5**).

**Table 1:** CD200R1 and CD200 expression on different cell types.

Cells	CD200R	CD200
Monocytes (*n* = 5)	8.09 ± 4.69	2.53 ± 0.34
MoDCs (*n* = 5)	29.65 ± 7.61	1.26 ± 0.05
MDSCs (*n* = 5)	8.40 ± 1.11	1.27 ± 0.04
CD4 non-activated (*n* = 5)	69.71 ± 15.35	2.87 ± 0.43
CD8 non-activated (*n* = 5)	108.65 ± 20.14	2.11 ± 0.20
CD4 activated^a^ (*n* = 5)	42.25 ± 24.83	6.37 ± 2.74
CD8 activated^a^ (*n* = 5)	80.43 ± 31.47	2.77 ± 1.04

Cells were incubated with fluorescently labeled antibodies directed against CD200 and CD200R1, and MFI was determined by flow cytometry. Results are expressed as fold from unstained condition.

a Activated with anti-CD3/CD28 for 4 days.

**Figure 2:**
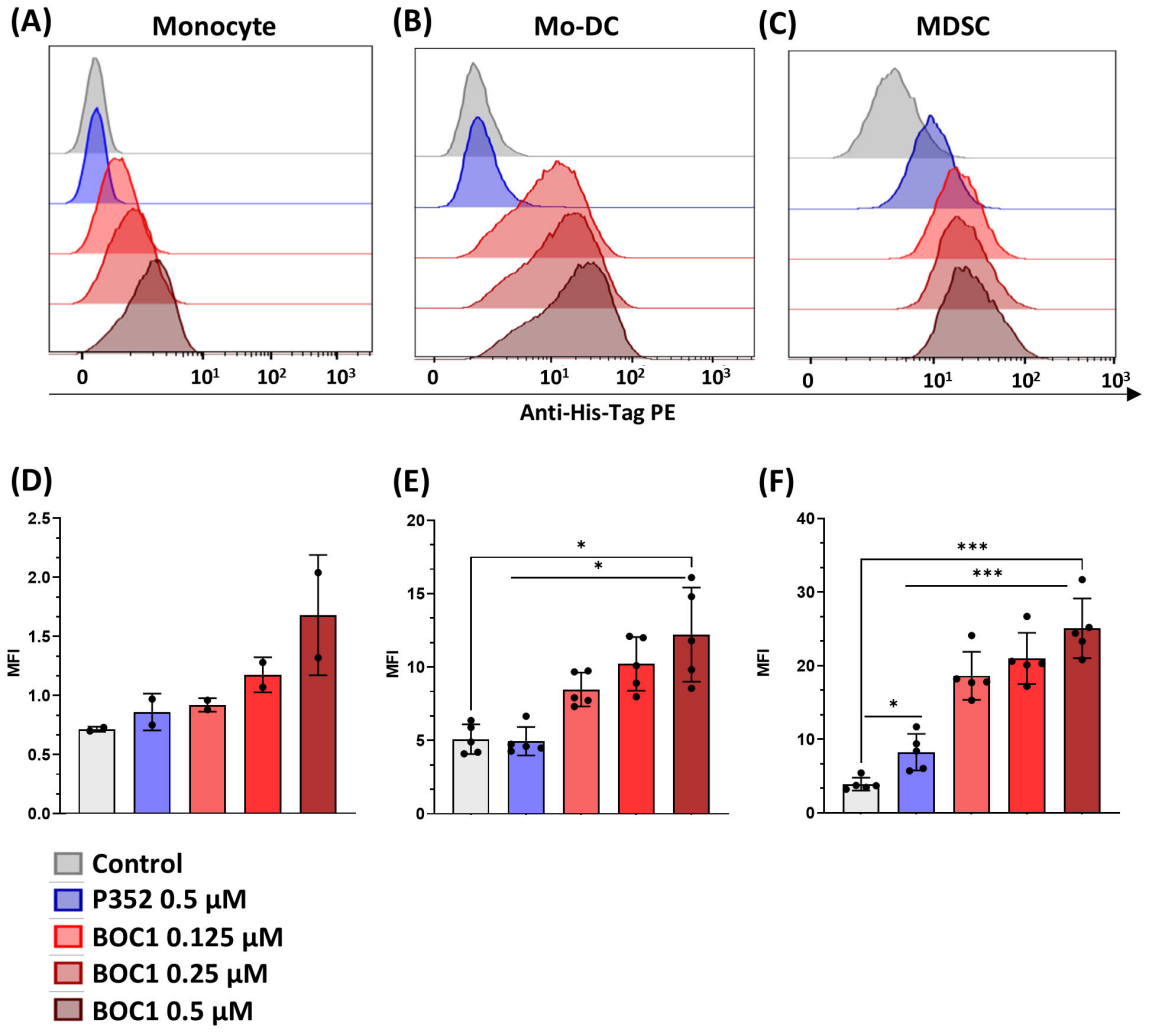
Protein binding to different cell types. Monocytes, MoDCs, or MDSCs were incubated with different concentrations (0.125, 0.25, 0.5 µM) of His-tagged BOC1, the control peptide P352 (0.5 µM), or without protein (control) for 1 h at 4°C, followed by PE-coupled anti-His-tag antibody addition. PE mean fluorescent intensity (MFI) was evaluated by flow cytometry. Representative histograms from one blood donor: monocyte **(A)**, MoDC **(B)**, or MDSC **(C)**. PE MFI of pool of two monocyte donors **(D)** or five MoDC **(E)** and MDSC **(F)** blood donors. Results represented as mean ± SD (monocytes) or mean ± SEM (MoDCs and MDSCs), paired *t*-test (ns, non-statistically significant; *p ≤ 0.05; ***p ≤ 0.0001).

In a second step, we analyzed BOC1 binding to human monocytes, MoDCs, and MDSCs by flow cytometry. The analysis was run testing different BOC1 concentrations (0.125, 0.250, 0.5 µM) followed by the addition of a PE-labeled anti-His-tag antibody, and the results, expressed as PE-MFI, were compared to those obtained using P352, a protein coming from the same library and used as a negative control, at the highest concentration (0.5 µM). While no or low binding (mainly on MDSCs, **Figures 2C–F**) was observed for P352, a consistent and dose-dependent binding was measured for BOC1 on the three cell types (**Figure 2**). As expected, weak BOC1 binding was observed on monocytes (**Figures 2A–D**). Despite the different CD200R1 expressions between MoDCs and MDSCs (**Table 1**), no disparity in BOC1 binding was highlighted between the two cell types (**Figure 2B, C, E, F**).

However, while these data clearly show BOC1 binding to cell types expressing high CD200R1 levels, we cannot definitively assess that the MFI shift observed in **Figures 2A–F** is the result of a direct interaction between BOC1 and CD200R1. We used esiRNA technology (Eupheria Biotech, Dresden, Germany) to generate CD200R1/KO monocytes and DCs (Eupheria Biotech, Dresden, Germany). In spite of the numerous conditions tested (esiRNA concentrations, different time points post-transfection, different cell types, and different validation methods such as qPCR and flow cytometry), we were not able to induce efficient CD200R1 knockdown, leaving open the possibility of alternative receptors/proteins contributing to the peptide’s efficacy.

### 3.4 BOC1 induces cytokine secretion by monocyte-derived dendritic cells

**Figure 3:**
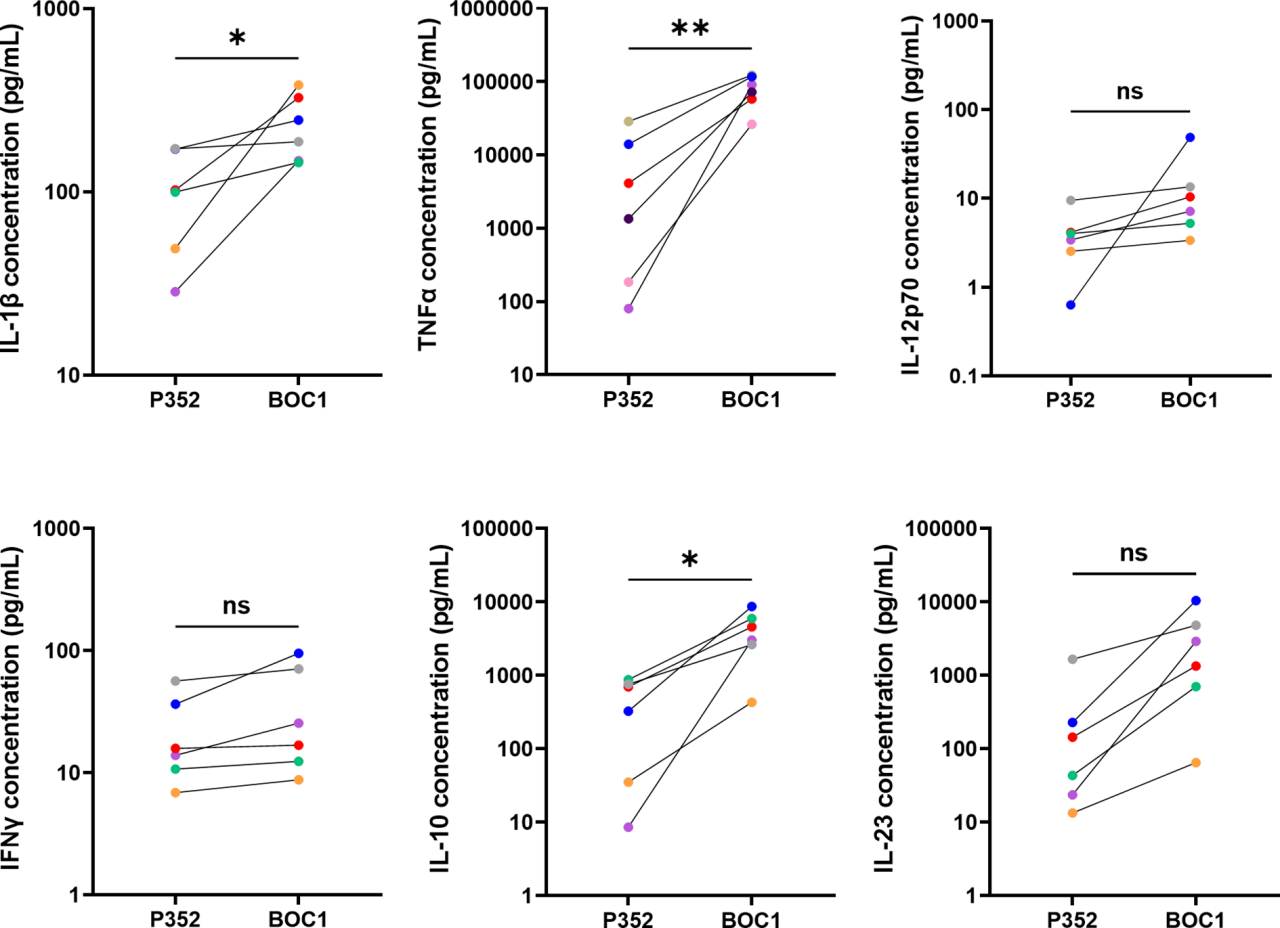
BOC1 induces cytokine secretion in MoDCs. Human MoDCs were incubated with 0.5 µM of BOC1 or the control peptide P352 for 24 h. Cytokines were quantified through LegendPLEX™ (IL-1β, IL-12-p70, IFNγ, IL-10, IL-23) or AlphaLISA (TNFα) technologies. Results (pg/mL) are from three independent experiments (six blood donors) and are represented as mean ± SEM, paired *t*-test (ns, non-statistically significant; *p ≤ 0.05; **p ≤ 0.001).

In accordance with previous results showing binding to MoDCs, we analyzed the impact of BOC1 on the induction of cytokine secretion. Cytokines were quantified in the supernatants of human MoDCs stimulated with BOC1 (0.5 µM) for 24 h. BOC1 induced the secretion of pro-inflammatory cytokines IL-1β and TNFα at levels approximately 2- and 10-fold greater, respectively, than those induced by the negative control protein P352 (**Figure 3**); low levels of IL-12p70 and IFNγ were secreted by MoDCs with no specific impact of BOC1. Interestingly, higher levels of IL-10 (~9-fold, *p* ≤ 0.05) and IL-23 (~9-fold, *p* = 0.1) were also observed. These results are extremely intriguing as contrasting functions are associated with these cytokines. However, activation and proliferation of antigen-activated intratumoral CD8^+^ T cells have been observed in cancer patients following treatment with pegilodecakin, a pegylated recombinant IL-10 (42, 43), and the intratumoral expression of IL-23 was proven to be effective in several tumor models (44).

### 3.5 BOC1 induces CD4 and CD8 proliferation in a MoDC–T-cell MLR

**Figure 4:**
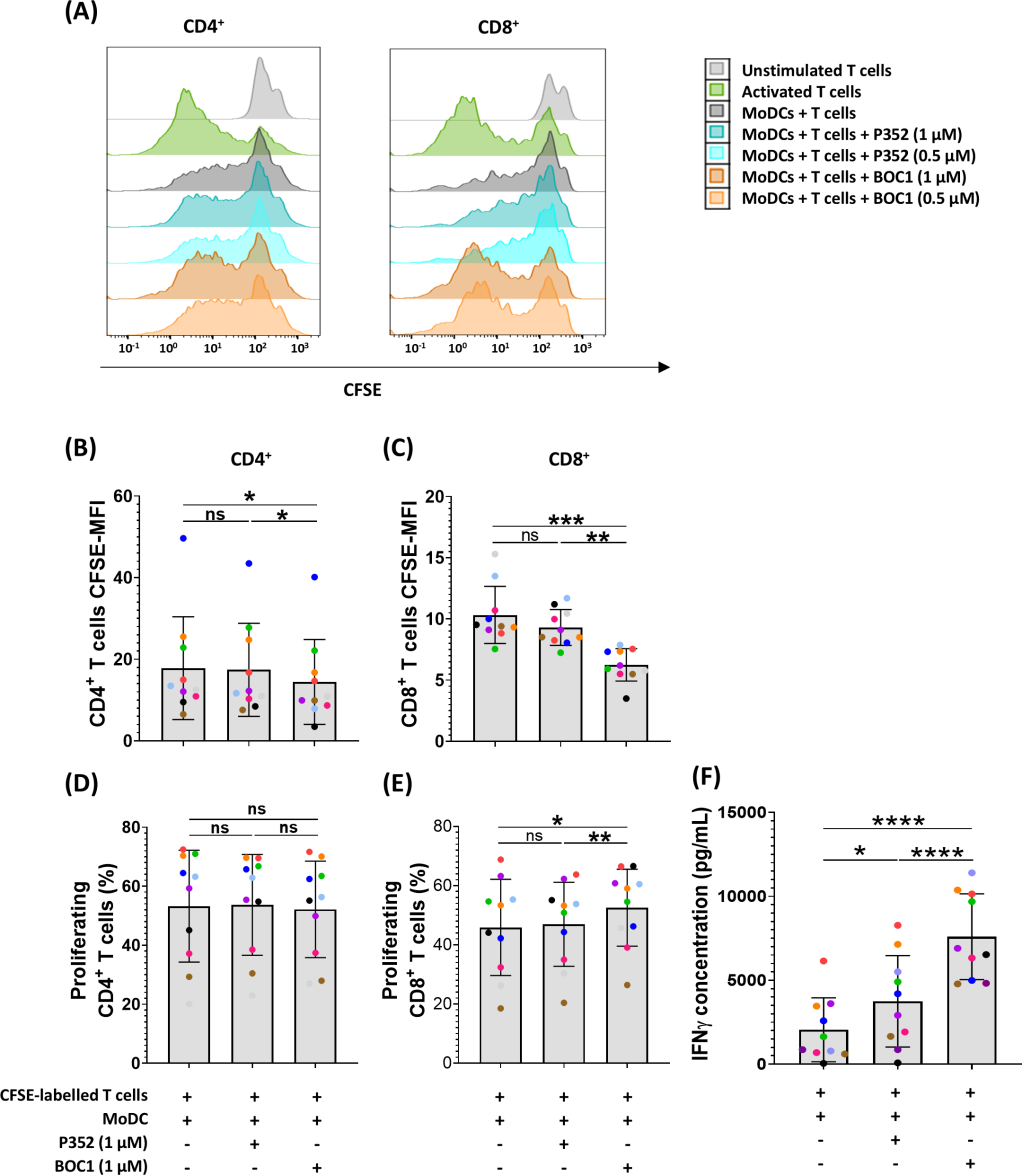
Induction of CD8^+^ proliferation and IFNγ secretion in a MoDC T-cell MLR. MoDCs, differentiated from monocytes during 7 days in the presence of GM-CSF (10 ng/mL) and IL-4 (10 ng/mL), were mixed with T cells (ratio 1:10) with and without BOC1 and P352. **(A)** Representative histograms showing CD4^+^ and CD8^+^ cell proliferation following treatment with 0.5 or 1 µM of BOC1 and P352. **(B)** CD4^+^ and **(C)** CD8^+^ proliferation expressed as CFSE MFI. **(D)** CD4^+^ and **(E)** CD8^+^ proliferation expressed as percentage of proliferating cells. **(F)** IFNγ concentration measured in MLR supernatants by AlphaLISA. Data in **(B–F)** show a pool of 10 T-cell–MoDC combinations of three independent experiments (T cells from four blood donors and MoDCs from five blood donors). Results are shown as mean ± SEM, paired *t*-test (ns, non-statistically significant; *p ≤ 0.05; **p ≤ 0.01; ***p ≤ 0.001; ****p ≤ 0.0001).

To explore BOC1 effectiveness as a potential candidate for immunotherapy, we investigated its capacity to increase CD8^+^ T-cell proliferation and IFNγ secretion following treatment of MoDCs in a classic allogenic MLR. To this goal, MoDCs were cocultured with T cells (1:10 ratio) with or without 0.5 or 1 µM of BOC1 or P352. Since a stronger immunomodulatory effect was observed following cell treatment with 1 µM of BOC1, this concentration was used for all the MLRs described in this section. As shown in **Figures 4A, B, D**, a weak proliferation of CD4^+^ T cells was induced by BOC1 with regard to untreated or P352-treated cells. Even though no difference in the percentage of proliferation was observed (**Figure 4D**), CFSE MFI was significantly lower (*p* ≤ 0.05) following cell treatment with BOC1(**Figure 4B**). A more significant proliferation of CD8^+^ T cells, considering both the percentage of proliferation (**Figure 4E**) and CFSE MFI (**Figure 4C**), was induced by BOC1 treatment compared to control conditions. The immunomodulatory effect of BOC1, underlined by flow cytometry analysis, was supported by significant (*p* < 0.01 and *p* < 0.001) IFNγ secretion (3.7- and 2-fold, respectively) in those cocultures treated with BOC1 toward those untreated or P352-treated (**Figure 4F**). We suppose that the moderate, donor-dependent increase of IFNγ induced by P352 may be due to residual contaminants present in the purified proteins.

### 3.6 BOC1 inhibits myeloid-derived suppressor cell differentiation

**Figure 5:**
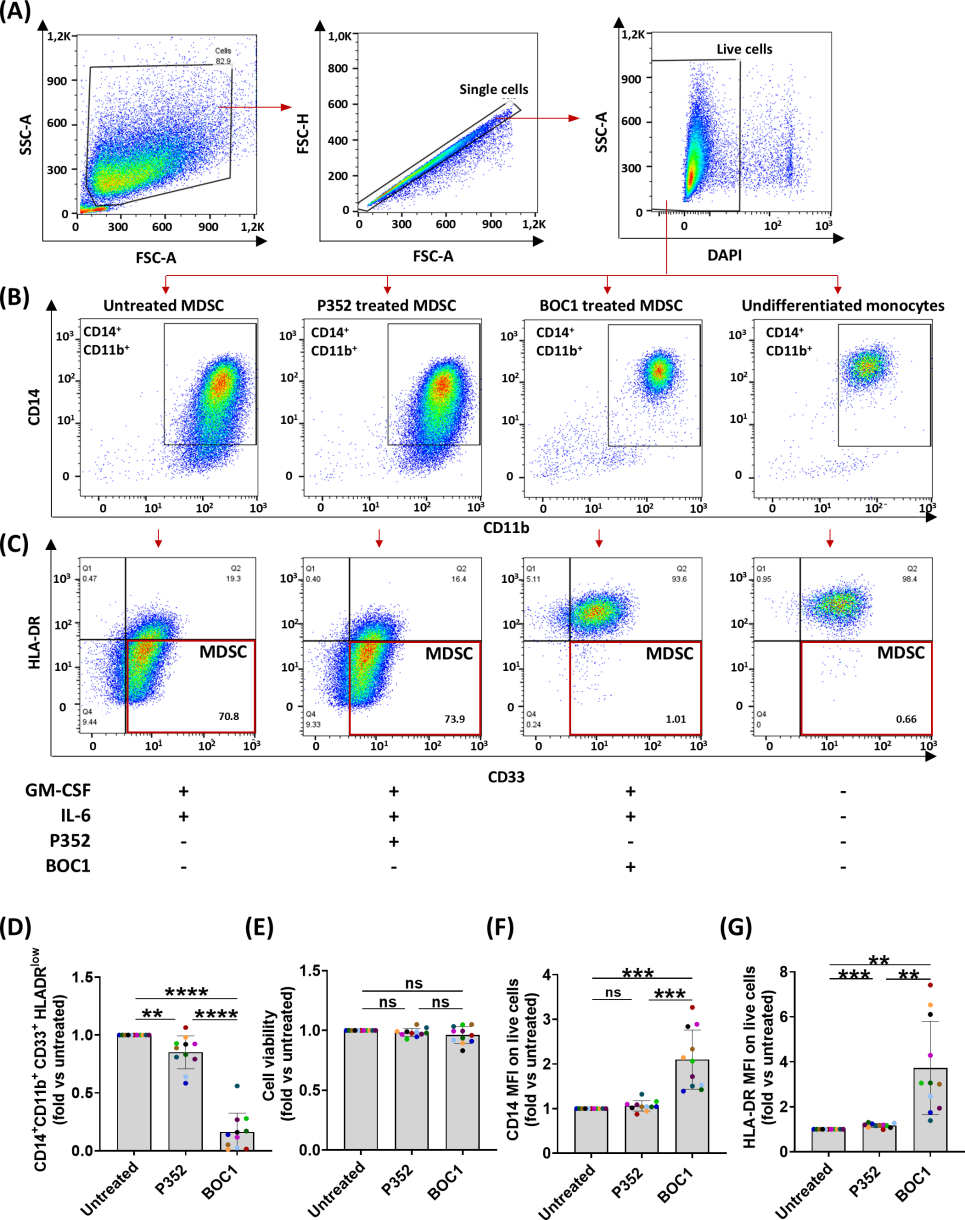
Inhibition of monocyte differentiation into MDSCs. Monocytes were differentiated in MDSCs by treatment with rhIL-6 and rhGM-CSF in the presence or absence of 10 nM of BOC1 or P352 for 7 days. **(A)** Gating strategy. **(B)** CD14^+^CD11b^+^ gating on live cells and **(C)** HLA-DR and CD33 expression on CD14^+^CD11b^+^ cells from untreated, P352- and BOC1-treated cells, or undifferentiated monocytes. **(D)** CD14^+^CD11b^+^CD33^+^ HLA-DR^low^-positive cells among live cells, **(E)** cell viability, and **(F)** CD14 and **(G)** HLA-DR MFI among CD14^+^CD11b^+^ cells, all represented as fold from the untreated group. Data **(A–C)** are representative histograms from one blood donor. Data **(D–G)** show a pool of 11 blood donors from six independent experiments. Results represented as mean ± SEM, paired *t*-test (ns, non-statistically significant; **p ≤ 0.01; ***p ≤ 0.0001; ****p ≤ 0.0001).

We further investigated the possible role of BOC1 in the TME; as previously mentioned, the CD200/CD200R1 axis has been described to be involved in MDSC differentiation and immunosuppressive function, promoting tumorigenesis (20, 23). Monocytic MDSCs are characterized by the CD14^+^CD11b^+^CD33^+^HLA-DR^low/−^ phenotype. To functionally assess the effect of BOC1 interaction with CD200R1, we evaluated the impact of BOC1 on monocyte differentiation into MDSCs. The protein BOC1 (or the negative protein P352) was added from the beginning of monocyte differentiation into MDSCs, together with rhIL-6 and rhGM-CSF (see details in the Experimental procedures), and the percentage of induced MDSCs was evaluated by flow cytometry by measuring the levels of CD33 and HLA-DR expressions within the CD14^+^CD11b^+^ population at the end of the differentiation process (**Figures 5A–C**). A short dose response of BOC1 (1, 10, 50 nM) was tested in a preliminary assay to determine the optimal concentration to be used (**Supplementary Figure 6**). BOC1 showed a trend on MDSC inhibition already at 1 nM and approximately 90% inhibition at 10 nM; thus, this concentration was selected for the functional validation of BOC1 (**Figure 5**). The MDSC population increased upon differentiation in the presence of IL-6 and GM-CSF (**Figure 5B**, far left panel, “Untreated” condition) compared to “Undifferentiated monocytes” (**Figure 5B**, far right panel). In the presence of 10 nM of BOC1 (“BOC1-treated” condition), we observed a striking decrease (~90%) of MDSC differentiation and proliferation with most cells showing a monocyte-like phenotype (CD14^+^CD11b^+^CD33^+^HLA-DR^+^, **Figures 5B–D**), characterized by higher CD14 and HLA-DR expression (2- and 4-fold, respectively) compared to the untreated cells (**Figures 5F, G**). On the contrary, no inhibitory effect on MDSC differentiation was observed in the presence of the negative peptide P352 (“P352-treated” condition) (**Figures 5B–D, F, G**). Importantly, no cytotoxic effect was observed in the presence of BOC1 or P352 proteins (**Figure 5E**). Altogether, these results suggest an antagonistic effect of BOC1 toward MDSC differentiation.

### 3.7 BOC1 restores CD4 and CD8 proliferation in an MDSC–T-cell MLR

Building on our previous findings regarding the ability of BOC1 to inhibit MDSC differentiation and the well-documented suppressive effects of MDSCs on T-cell proliferation (45, 46), we sought to investigate whether BOC1 treatment of monocytes could enhance T-cell activity compared to untreated cells. To test this hypothesis, we performed MLR, where monocytes differentiated into MDSCs were cocultured with allogenic T cells. This experimental setup allowed us to evaluate how BOC1 influences the ability of MDSCs to impair T-cell proliferation.

**Figure 6:**
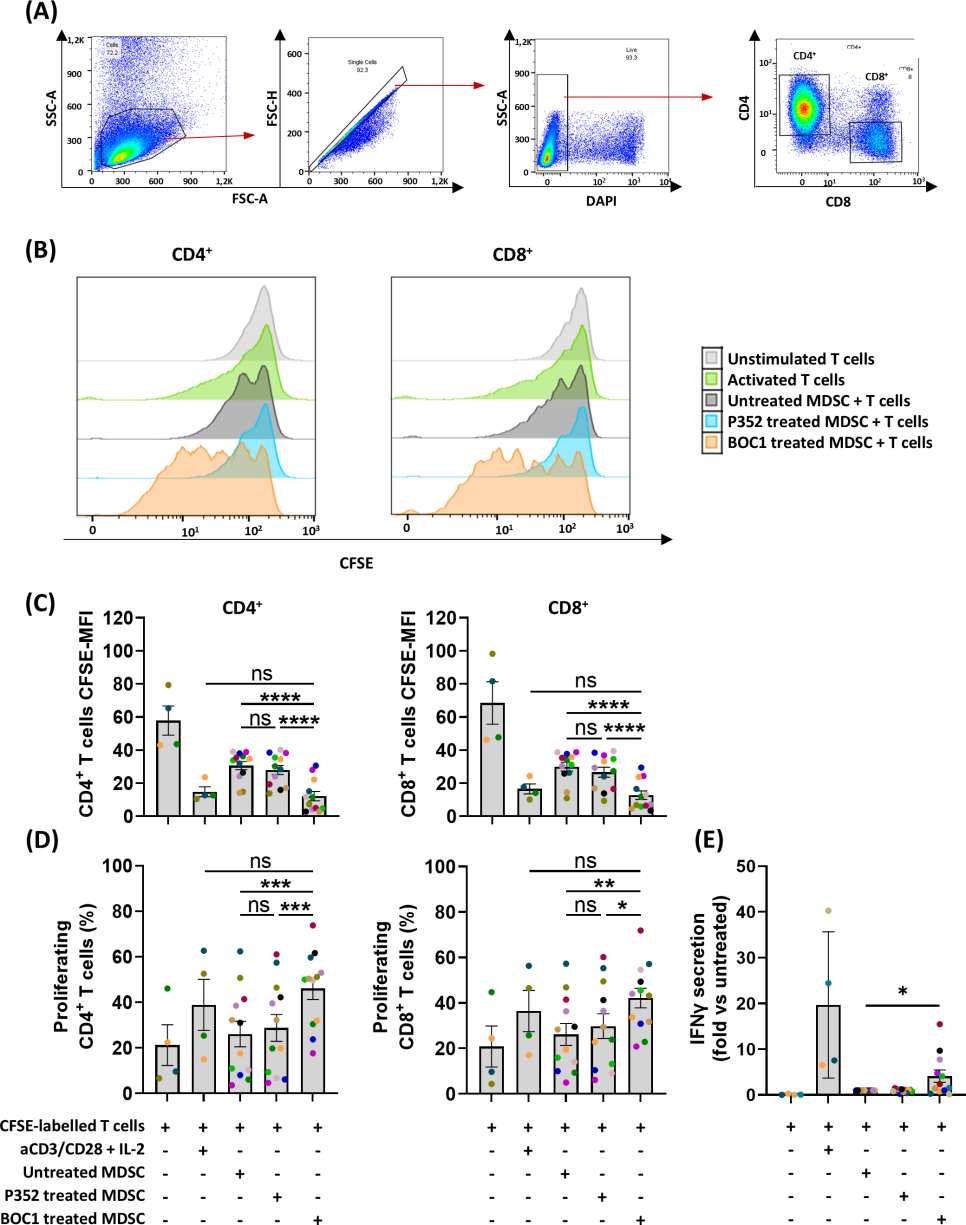
Restoration of CD4^+^/CD8^+^ proliferation in MDSC T-cell suppression assay. MDSCs, untreated or pretreated (during differentiation) with 10 nM of BOC1 or P352 during 7 days, were cultured with allogenic CFSE-labeled T cells (ratio 1:4) for 5 days. **(A)** Gating strategy. **(B)** Representative histograms showing CD4^+^ and CD8^+^ cell proliferation following BOC1 (orange) suppressive effect on MDSCs. Similar pattern between untreated MDSCs (gray) and P352-treated MDSCs (light blue). **(C)** CD4^+^ and CD8^+^ CFSE MFI measured from unstimulated or aCD3/CD28-activated T cells or T cells cultured with P352- or BOC1-treated MDSCs. **(D)** CD4^+^ and CD8^+^ percentage of proliferating cells under the different culture conditions. **(E)** IFNγ secretion in supernatant of MDSC T-cell coculture. Data **(B)** are representative of one blood donor. Data (**C–E**) show a pool of 12 MDSC–T-cell combinations of two independent experiments (T cells from four blood donors and monocytes/MDSCs from six blood donors). Results are shown as mean ± SEM, paired *t*-test (ns, non-statistically significant; *p ≤ 0.05; **p ≤ 0.01; ***p ≤ 0.001; ****p ≤ 0.0001).

Monocytes, grown under MDSC differentiation conditions with or without 10 nM of BOC1 or P352 for 7 days, were harvested and then cultured with allogenic CFSE-labeled T cells (ratio 1:4) for 5 days. Proliferation of the CFSE-labeled T cells was analyzed by flow cytometry within the DAPI^−^CD3^+^ population, for both CD4^+^CD8^−^ and CD4^−^CD8^+^ subpopulations, by measuring the decrease of CFSE MFI during cell division (**Figure 6**). Unstimulated and aCD3/CD28-stimulated T cells were considered the negative and positive controls of cell proliferation, respectively. As expected, while non-stimulated CD4^+^CD8^−^ and CD4^−^ CD8^+^ T cells showed high CFSE fluorescent intensity, aCD3/CD28 stimulation induced T-cell proliferation, evidenced by an increase in the percentage of proliferating cells and a reduction in CFSE fluorescent intensity (**Figures 6B–D**). Coculture of CFSE-labeled allogenic T cells with MDSCs showed high CFSE MFI, at a similar rate as unstimulated T cells, although a proliferation peak was observed in some cases (shown in the representative **Figure 6B**). A similar profile was observed for T cells cultured in the presence of P352-treated cells. Consistently, when T cells were cocultured with BOC1-treated cells, a statistically significant increase in cell proliferation (evidenced by reduced CFSE MFI and increased percentage of proliferating cells) was observed for both CD4^+^CD8^−^ and CD4^−^CD8^+^ T cells (**Figures 6B–D**). Moreover, IFNγ quantified in supernatants of the MDSC–T-cell cocultures was found significantly increased (4-fold) when BOC1-treated cells were used compared to both untreated and P352-treated cells (*p* ≤ 0.05) (**Figure 6E**). Collectively, these results indicate that BOC1 largely contributes to enhancing T-cell proliferation in an immunosuppressive MDSC-differentiating environment.

### 3.8 CD200 and BOC1 may bind to different regions of CD200R1

**Figure 7:**
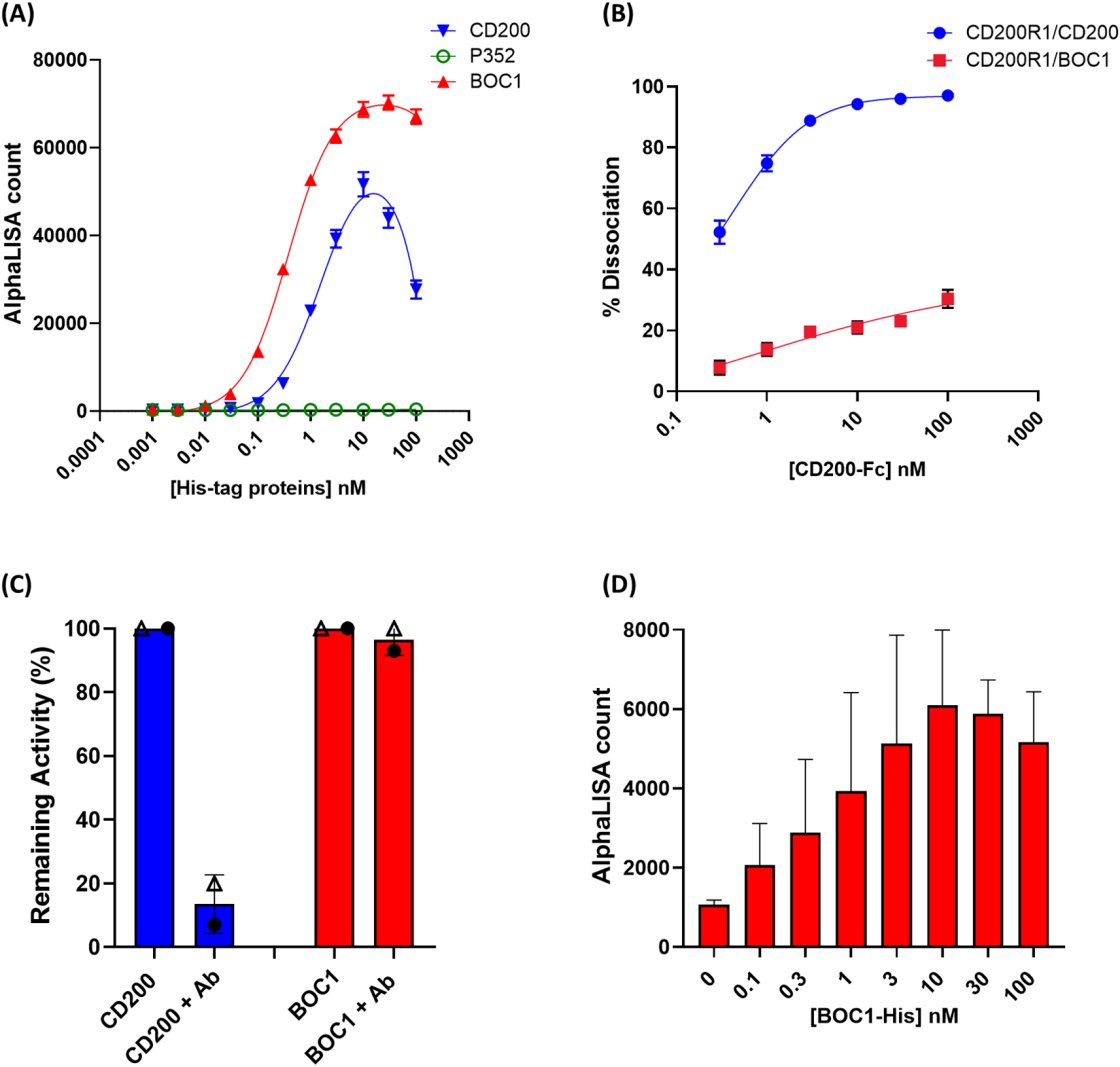
Protein–protein interaction and competition measured by AlphaLISA™. **(A)** Interaction between 3 nM of CD200R1-(biot) and a dose response (0.1 to 100 nM) of CD200-His (blue line), BOC1 (red line), and P352 (green line), measured as AlphaLISA count. **(B)** Dissociation (%) of the CD200R1-(biot)/CD200-His complex (0.3 nM of CD200R1-(biot) and 3 nM of CD200-His) (blue line) or the CD200R1-(biot)/BOC1-His complex (red line) by a dose response (0.1 to 100 nM) of CD200-Fc. **(C)** Dissociation (%) of the CD200R1-(biot)/CD200-His complex (blue bar, each protein is used at 3 nM) or the CD200R1-(biot)/BOC1-His complex (red bar, proteins at 3 nM) by an anti-CD200R1 antibody (30 nM). Data are first normalized vs. the background (AlphaLISA beads only), then expressed as remaining activity, and the % of complex inhibition is indicated. **(D)** Competition of CD200R1-(biot)/CD200-Fc (0.3 nM of CD200R1-(biot) and 3 nM of CD200-Fc) complex using a dose response (0.1 to 100 nM) of BOC1-His. Data are expressed as the AlphaLISA signal generated by the interaction between CD200R1-(biot) and the BOC1-His. Data reported **(A, B)** represent the mean of three independent experiments (each experiment including four replicates/point). Error bars represent the SEM. Data reported **(C, D)** represent the mean of two independent experiments (each experiment including four replicates/point). Error bars represent the SD.

Despite the similarity existing between CD200 and CD200R1 (the latter supposed to come from duplication of the CD200 gene) (41), no similarity in either the amino acid sequence or the three-dimensional structure could be highlighted between BOC1 and CD200 or CD200R1 proteins. Thus, a deep characterization of BOC1–CD200R1 interaction was run to get more insights into BOC1’s mechanism of action. BOC1-His (hereafter called BOC1) was found to interact with 3 nM of CD200R1-(biot) (hereafter called CD200R1) in a dose-response manner (**Figure 7A**), yielding a slightly higher AlphaLISA signal (*B*_max_, **Table 2**) compared to the same titration of CD200-His (hereafter called CD200). Interestingly, the *K*_D_ generated by the BOC1/CD200R1 complex was four times lower than that produced by the CD200/CD200R1 complex (**Table 2**), demonstrating a higher affinity of BOC1 than CD200 toward CD200R1. Importantly, no signal was generated, in any of the tested concentrations, by P352-His. A dose response (0.1 to 100 nM) of untagged CD200 (CD200-Fc) was, as expected, able to interfere with the CD200/CD200R1 complex, reaching 93% complex dissociation at 10 nM concentration. Surprisingly, the same concentration of CD200-Fc induced only minimal dissociation (~20%) of the BOC1/CD200R1 complex, and a slight positive dissociation trend was observed at higher concentrations (approximately 30% dissociation at 100 nM) (**Figure 7B**). Similarly, a human anti-CD200R1 antibody (30 nM) strongly impacted the interaction of CD200/CD200R1 (reaching 98% inhibition) but failed to turn off the AlphaLISA signal generated by BOC1/CD200R1 interaction (**Figure 7C**). We could not directly assess the ability of BOC1 to compete with CD200 binding to CD200R1 using the same setup because of its His-tag (elimination of the tag on purified proteins is technically possible but restricted by the high protein loss following tag digestion and protein repurification). Thus, we set up a similar test by incubating CD200-Fc with CD200R1 and using BOC1 as a potential competitor. As shown in **Figure 7D**, a growing AlphaLISA signal was generated by increasing BOC1 concentrations, suggesting that the presence of CD200 does not prevent BOC1 binding to CD200R1. Alternatively, the results from **Figures 7B, C** support the hypothesis of different anchor points of BOC1 and CD200 on CD200R1.

**Table 2:** *B*_max_ and *K*_D_ determined for CD200-His and BOC1-His binding to CD200R1-(biot).

Kinetic Parameter	CD200	BOC1	P352
***B*_max_** (AlphaLISA counts)	60,846 ± 3,720	72,551 ± 2,035	–
***K*_D_** (nM)	1.56 ± 0.30	0.38 ± 0.04	–

His-tagged proteins were used between 0.1 and 100 nM. CD200R1-(biot) was used at 3 nM. Data, representing the mean of two independent experiments, were analyzed by GraphPad PRSIM using the equation “One site-total.”. Bmax, maximum binding capacity; KD, equilibrium dissociation constant.

### 3.9 BOC1 AlphaFold structure prediction and proposed binding to CD200R1

**Figure 8:**
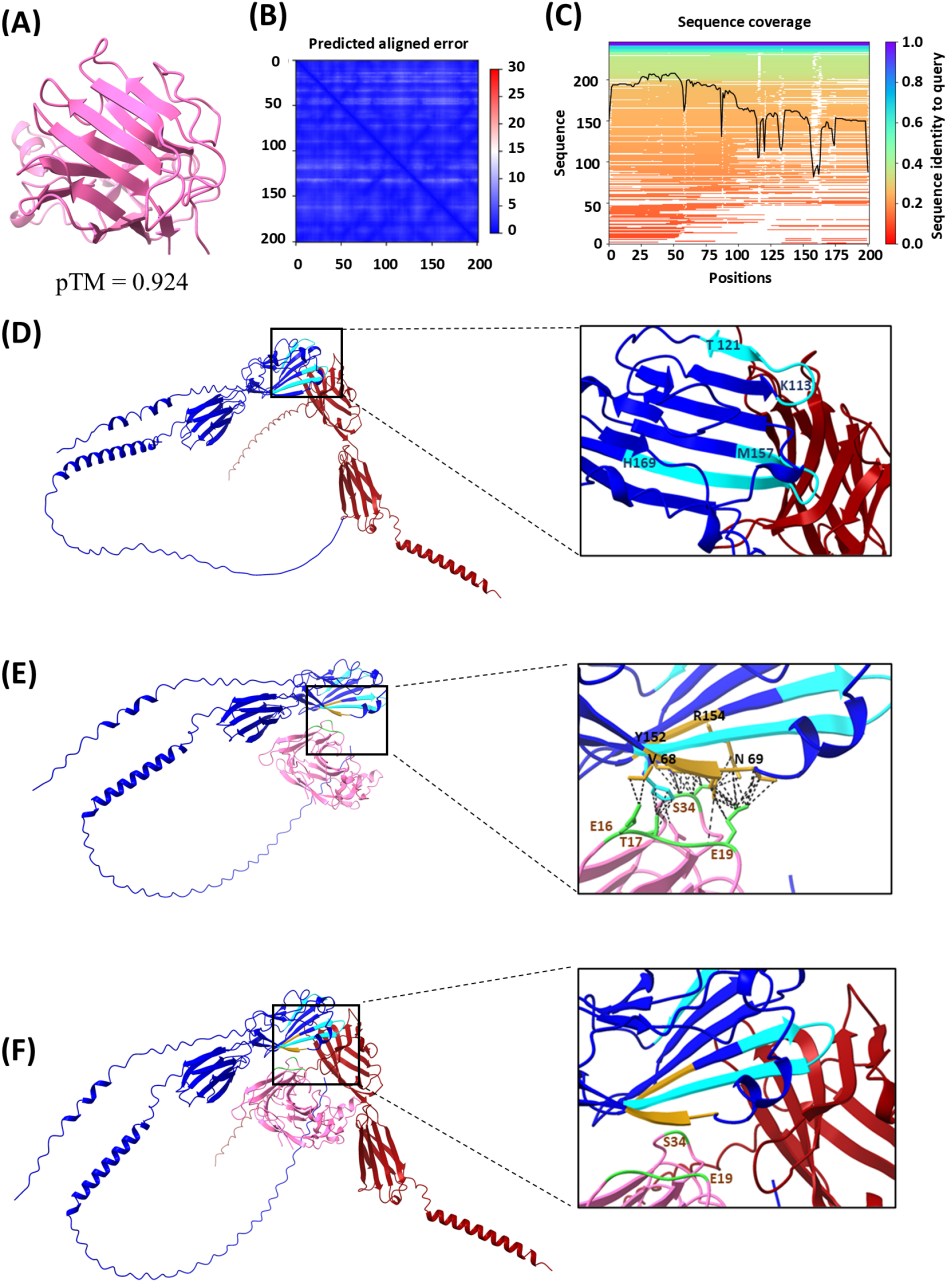
Protein structure and protein–protein interaction modeling results. Analysis was performed using the AlphaFold Server powered by AlphaFold 3 and UCSF ChimeraX 1.8 software. **(A)** BOC1 structure prediction (best model), with a predicted template modeling (pTM) score of 0.924. **(B)** Local distance difference test (pLDDT) and predicted aligned error (PAE), atom b factor range 87.1 to 98.8. **(C)** Sequence coverage. **(D)** CD200R1 (blue) and CD200 (dark red) interaction prediction, with relevant regions of CD200R1 for this interaction indicated in light-blue CDR2 between positions 113 and 121 (KETNETKET), and CDR3 between positions 157 and 169 (MVTPDGNFHRGYH). **(E)** CD200R1 (blue) and BOC1 (pink) predicted interaction, with relevant amino acids on BOC1/CD200R1 interaction indicated in golden yellow on CD200R1 (V68, N69, Y152, and R154) and in green on BOC1 (mainly E19 and S34, but also E16 and T17), and the predicted contacts indicated as black dashed lines. **(F)** Merge of **(D, E)** (using ChimeraX 1.8 Matchmaker tool).

The intriguing hypothesis of different anchor points of BOC1 and CD200 on CD200R1 was corroborated by AlphaFold prediction (AlphaFold Server powered by AlphaFold 3 and UCSF ChimeraX for visualization) (47). We first evaluated BOC1 structure prediction and then compared the prediction of CD200R1/BOC1 interaction with that established for the CD200/CD200R1 complex (28, 48). BOC1’s best folding prediction is shown in **Figure 8A** [pLDDT with a pTM score of 0.924 and the PAE (**Figure 8B**) and the sequence coverage (**Figure 8C**) are shown as well]. Overall, the high coverage for most of the sequence, the low PAE, and the high pTM indicate a confident high-quality structure prediction.

**Figure 8D** shows the interaction between CD200 (dark red) and CD200R1 (blue); CD200R1 regions described to be relevant for this interaction (28) are indicated in light blue. Both CD200R1-relevant regions [CDR2 between amino acids 113 and 121 (KETNETKET) and CDR3 between amino acids 157 and 169 (MVTPDGNFHRGYH)] are located in the N-terminal region and considered crucial for the interaction between the two proteins. The predicted interactions between BOC1 (pink) and CD200R1 (blue) are shown in **Figure 8E**. On the zoomed image are highlighted the amino acids from both CD200R1 (golden-yellow) and BOC1 (green) that showed the highest proportion of interaction (intramodel contacts with a center-to-center distance ≤4.0Å generated by UCSF ChimeraX). As observed, even if the predicted region of CD200R1 interacting with BOC1 stays within the N-terminal region of CD200R1, it does not exactly match the interactions described for the CD200/CD200R1 complex (49). Specifically, E19 from BOC1 is predicted to interact with V68 and N69 of CD200R1, while S34 from BOC1 is expected to interact with Y152 and R154 of CD200R1, in proximity to the CDR3 region. Furthermore, T17 from BOC1 is predicted to interact with H169, which is the terminal amino acid of the CDR3 region.

These computational predictions are consistent with the results from competition assays (**Figures 7B–D**). In these assays, we observed minimal competition when increasing concentrations of CD200-Fc were added to the BOC1/CD200R1 complex, resulting in only approximately 30% dissociation at 100 nM (**Figure 7B**). Additionally, no impact was observed when incubating a fixed CD200-Fc concentration with biotinylated CD200R1 and increasing concentrations of BOC1-His (**Figure 7D**).

Furthermore, when merging both protein–protein interaction predictions (**Figure 8F**), we confirm the spatial proximity of BOC1 and CD200 in their interaction with CD200R1, while no substantial overlap in their binding interfaces is observed.

### 3.10 The full-length protein is necessary for proper interaction with CD200R1

**Figure 9:**
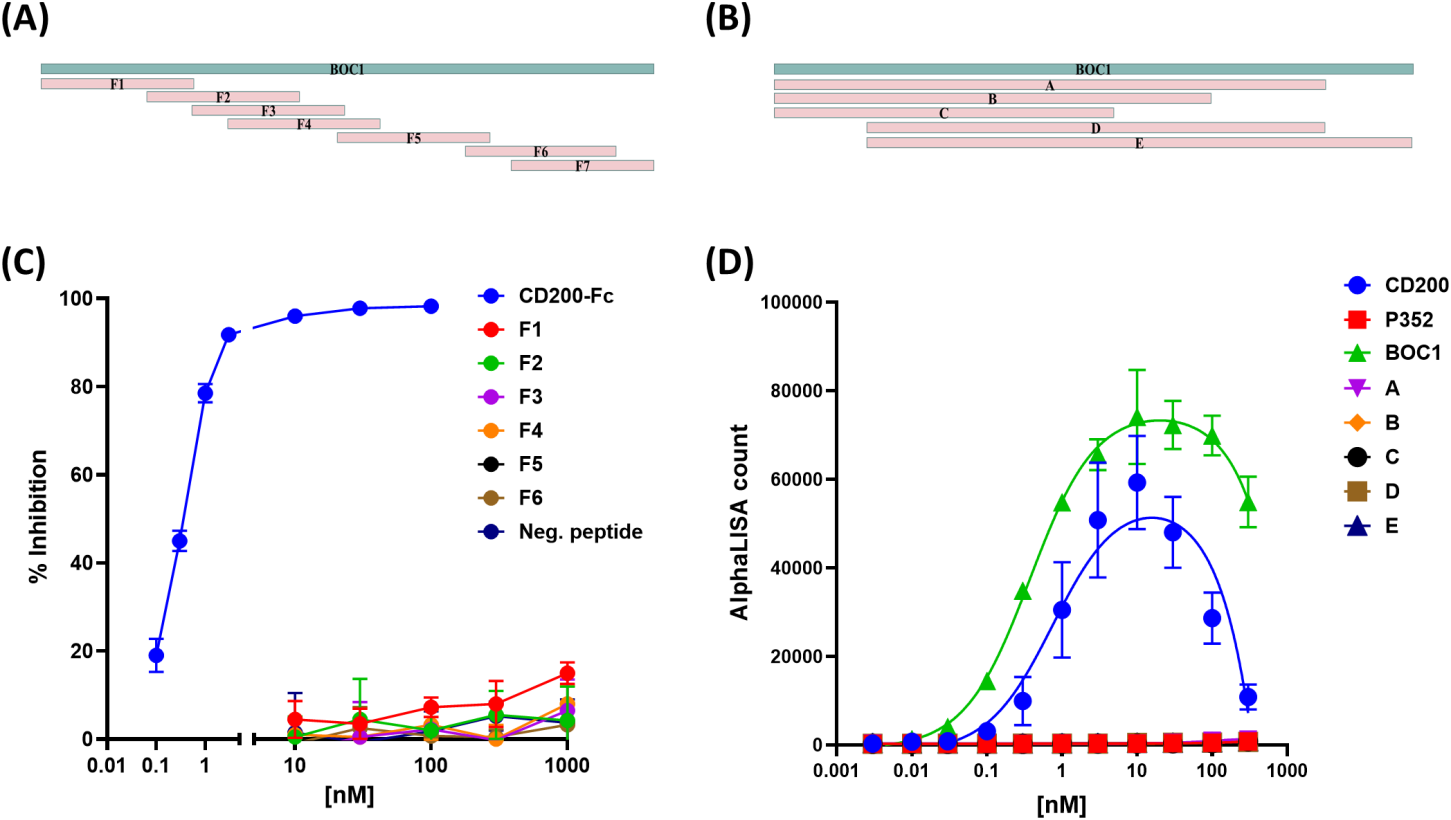
Effect of short BOC1 sequences on the interaction with CD200R1. Design of 50 aa-long **(A)** or >100 aa-long **(B)** peptides covering the BOC1 sequence. **(C)** Competition assay of CD200-(His)/CD200R1-(biot) (3 nM each) interaction by 50 aa-long peptides used between 10 and 1,000 nM. Untagged CD200 (CD200-FC, blue line) is used (0.1−100 nM) as a positive control. **(D)** Interaction between 3 nM of CD200R1-(biot) and a dose response (0.1 to 100 nM) of His-tagged CD200 (blue dots) or BOC1 (green triangles) or long (>100 aa) BOC1 fragments. Data **(C)** are expressed as % inhibition of the CD200/CD200R1 complex and are shown as mean ± SD of quadruplicate measurements of a representative of two independent experiments. Data **(D)** are expressed as AlphaLISA signal produced by protein interactions and are shown as mean ± SD of quadruplicate measurements of a representative of two independent experiments.

With the aim of finding the minimal region of BOC1 responsible for the interaction with CD200R1, a set of 50 aa-long untagged overlapping peptides, covering the full-length sequence (**Figure 9A**), was produced by chemical synthesis, and a large range of concentrations of each peptide (from 0.01 µM to 1 µM) was tested by AlphaLISA™ for their capacity to disturb the CD200/CD200R1 interaction. None of the tested peptides was able to reverse, at any of the concentrations used, the positive signal obtained following the interaction between CD200/CD200R1 (**Figure 9C**). Thus, longer (>100 aa) overlapping sequences were designed (**Figure 9B**), and the respective His-tagged proteins were tested between 0.1 and 100 nM as binders of CD200R1 (3 nM). No positive signal was measured for any of the BOC1 variants (**Figure 9D**), meaning that the full-length protein is necessary to get the optimal 3D conformation required for interacting with CD200R1.

### 3.11 BOC1 mutant analysis

**Figure 10:**
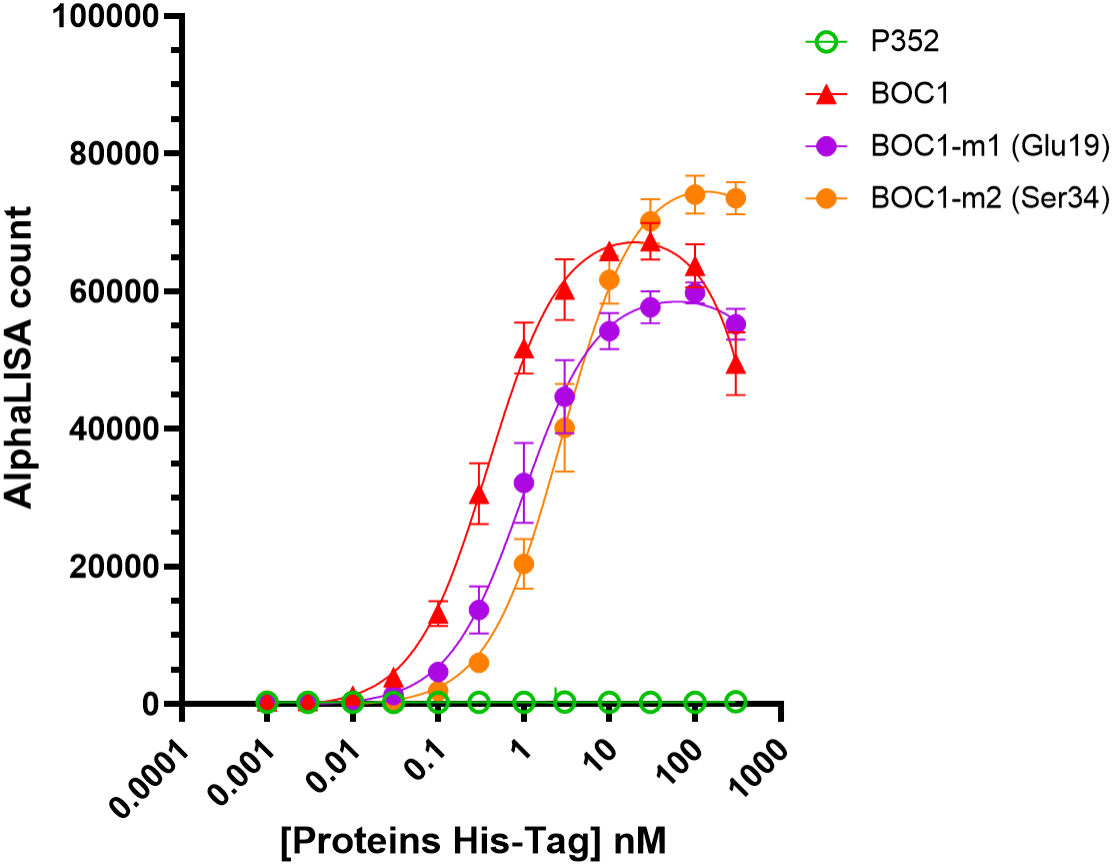
BOC1 mutants’ interaction with CD200R1 measured by AlphaLISA™. Interaction between CD200R1-(biot) (3 nM) and a dose response (0.1 to 300 nM) of BOC1-m1 (Glu19, purple circles) and BOC1-m2 (Ser34, orange circles) mutants, measured as AlphaLISA count. BOC1 (red triangles) and P352 (green open circles) are used as positive and negative controls, respectively. Data represent the mean of two independent experiments (each experiment including four replicates/point). Error bars represent the SD.

According to AlphaFold analysis, the N-terminal region of BOC1 might be important for the interaction with CD200R1. Notably, Glu19 and Ser34 were highlighted in all top 5 predictions provided by AlphaFold (not shown) and, for that reason, chosen as candidate amino acids for site-directed mutagenesis. Mutants for each amino acid were produced with an alanine replacing each target residue, and their capacity to form a complex with CD200R1 was evaluated by AlphaLISA™. Both mutants were able to generate an AlphaLISA signal following interaction with CD200R1 but with lower (2.5- and 7-fold, respectively) affinity, with regard to the unmutated protein (**Figure 10**; **Table 3**). Besides agreeing with the AlphaFold prediction, these results highlight the importance of the N-terminal region of BOC1 for binding to CD200R1 and suggest that the overall conformation is required to allow this interaction. However, a more complete mutant study, encompassing the full BOC1 N-terminal region, should be performed to identify all the key amino acids necessary for the interaction with CD200R1.

**Table 3:** *B*_max_ and *K*_D_ values determined for BOC1 and its mutants binding to CD200R1.

Kinetic Parameter	CD200	P352	BOC1	BOC1-m1 (Glu19)	BOC1-m2 (Ser34)
***B*_max_** (AlphaLISA counts)	52,062 ± 4,315	NA	70233 ± 1,576	60,528 ± 1,733	77,906 ± 2,094
***K*_D_** (nM)	0.95 ± 0.27	NA	0.38 ± 0.04	0.96 ± 0.12	2.78 ± 0.27
***B*_max_/*K*_D_**	54,796.34	NA	183,952.33	62,886.23	28,074.23

His-tagged proteins were used between 0.1 and 100 nM. CD200R1-(biot) was used at 3 nM. Data, representing the mean of two independent experiments, were analyzed by GraphPad PRISM using the equation “One site-total.”. Bmax, maximum binding capacity; KD, equilibrium dissociation constant.

## 4 Conclusion

Comprehensive scientific literature has highlighted the relevance of gut microbiota on human health. Thanks to metagenomic studies, researchers have featured links between the presence and abundance of bacteria and bacterial genes with the predisposition to develop inflammatory, metabolic disorders (50–52) and also cancer (53). Beyond pathogenesis, bacteria and bacteria-derived compounds have been described for their anti-inflammatory effect *in vitro* and *in vivo* (54–56), and an intriguing role in enhancing the outcome of anti-CTLA-4 and anti-PD-L1 immunotherapy has also been highlighted (30, 31, 57). While therapies based on ICI are a key interest in the treatment of cancer, a moderate percentage of patients positively respond to these treatments, and the need for new targets or drugs, enhancing their action and safety, arises. CD200R1 is a key inhibitory receptor expressed primarily on myeloid cells, including monocytes, macrophages, dendritic cells, and MDSCs, as well as on T cells (41). This receptor is involved in strong immune suppression via interaction with CD200 and has become the target of numerous investigations (14, 58, 59). CD200/CD200R1 interaction was first evaluated as a natural treatment regulating various autoimmune disorders (15–18), but later associated with MDSC and regulatory T-cell (Treg) differentiation in the TME, leading to the suppression of antitumor immunity (19, 20). Consequently, CD200/CD200R1 blockade has recently been considered a potential target in some forms of cancer. CD200/CD200R1 interaction occurs through cell-to-cell contact but also through free CD200 released following ectodomain shedding by metalloproteases. This last point is of particular interest since interaction with CD200R1 does not imperatively require cell presentation but can be achieved by circulating proteins. Myriads of secreted bacterial proteins are disseminated in the gut and represent the main mechanism used by bacteria to interact with their host. Finding adapted criteria of protein selection for library assembling, together with the development of specific screening tools, is crucial for the identification of potential future drugs (60). Sec pathway is the canonical pathway used by bacteria to transport unfolded proteins on the cell surface or outside the cell (61). It specifically recognizes a small amino acid sequence at the N-terminal region of a protein, called “signal peptide,” which is typical of secreted proteins and which we used as the preliminary criteria of protein selection. Next, we developed a PPI assay based on the AlphaLISA™ technology that was specific for the identification of proteins positively interacting with CD200R1 and that allowed us to identify and characterize the BOC1 protein. Despite bearing a similar *B*_max_ value as CD200, BOC1 showed 4-fold higher affinity than the natural ligand when forming a complex with CD200R1 (**Table 2**). This result suggests that BOC1 might disturb CD200/CD200R1 interaction. Although a classic competition assay (using BOC1 as a competitor of CD200/CD200R1 interaction) was not possible due to the AlphaLISA™ setting, we showed that CD200-Fc poorly affected BOC1/CD200R1 interaction (20% complex dissociation observed at 10 nM) while inducing 93% dissociation of the CD200/CD200R1 complex (**Figure 7B**). These results, together with the observation that an anti-CD200R1 antibody selectively disrupted CD200/CD200R1 interaction while leaving BOC1/CD200R1 interaction intact, have raised questions regarding the specific anchor points of BOC1 and CD200 on CD200R1. Important regions for interactions with CD200 (CDR2 and CDR3) are located in the N-terminal of CD200R1 (28). Since no sequence homology was found between CD200 and BOC1, we applied AlphaFold analysis to get predictions of BOC1 folding and interaction with CD200R1. Although predicted interactions between BOC1 and CD200R1 do not match those described for the CD200/CD200R1 complex, they still stay in the N-terminal region of CD200R1 (**Figures 8E, F**) as demonstrated by reduced affinity generated by BOC1 Glu19 and Ser34 mutants (**Figure 10**). The immunomodulatory potential of BOC1 was first evaluated on MoDCs, where the protein positively affected the secretion of IL-1β, TNFα, IL-10, and IL-23 without impacting those of IL-12p-70 or IFNγ (**Figure 3**). While the presence of IL-1β and TNFα is desirable in the TME to augment the activity of cytotoxic T cells, the concurrent increase of IL-10 and IL-23 might raise questions since these cytokines are often associated with the promotion of a tolerogenic environment or in fostering tumor growth and metastases. However, contrasting results indicate a positive outcome of these cytokines in immunotherapy as reported for pegilodecakin, a pegylated recombinant IL-10, tested alone or in combination with anti-PD-1 in patients with advanced renal carcinoma (43), or in combination with FOLFOX (folinic acid, fluorouracil, oxaliplatin) in metastatic pancreatic cancer patients (62). Preclinical data have also revealed the positive effect of the intratumoral expression of IL-23 in several tumor models (44). Secondarily, we evaluated the immunostimulant potential of BOC1 by classic MoDC:T-cell MLR (**Figure 4**) and showed that BOC1, but not P352, was able to induce T-cell proliferation and IFNγ secretion. The binding of CD200 to CD200R1 has been shown to strongly compromise immune response *in vitro*, by suppressing T-cell proliferation and IFNγ secretion while enhancing the MDSC population (49).

MDSCs represent a diverse group of underdeveloped myeloid cells that possess potent immune-inhibiting capabilities. During tumor growth, these cells experience a dramatic increase in number, proliferating extensively throughout the body. The CD200/CD200R1 axis has been shown to be involved in the expansion of the MDSC population and tumor proliferation, and different studies have suggested that inhibition of this axis could enhance the efficacy of immunotherapy (20, 29). Here, we hypothesized that compounds interfering with CD200/CD200R1 complex formation should restore T-cell proliferation and cytokine production. No bacterial effector with these properties has been identified so far; to date, only antibodies are used as ICI. Samalizumab is the only antibody described for blocking CD200 binding to CD200R1, and results showing the positive outcome of 23ME-00610 antibody blocking CD200R1 in patients with advanced solid malignancies have just been published (27). We got impressive results when testing BOC1 on MDSC differentiation. Used at 10 nM, BOC1 drastically reduced (~90%) MDSC differentiation (**Figure 5**), maintaining cells in an immature (monocyte-like) stage (CD14^+^CD11b^+^CD33^+^HLA-DR^+^). The observed effect was specific to BOC1 as another protein (P352), used as a negative control, failed to affect the MDSC phenotype. By reducing the MDSC population, BOC1 was effective in restoring CD4^+^/CD8^+^ proliferation and IFNγ secretion in an MDSC:T-cell suppressive assay (**Figure 6**). As CD8^+^ T cells and IFNγ are crucial mediators in fighting cancer, these results support the hypothesis that BOC1 may alter the tumor microenvironment, making it hostile to tumor growth. Despite the exceptional immunoregulatory effects observed *in vitro* with BOC1, we were unable, under the experimental conditions employed, to induce any CD200R1 knockdown in monocytes or dendritic cells, a necessary step to validate our proposed direct interaction between CD200R1 and BOC1. Thus, further research is necessary on one side to elucidate the mechanism of action (using either CRISPR-Cas9 or surface plasmon resonance) and on the other side to validate its potential therapeutic application *in vivo*.

Considering both the AlphaLISA and the *in-vitro* data, we speculate that BOC1 behaves as an ICI preventing CD200 binding to CD200R1, and we propose a mechanistic hypothesis wherein BOC1 binding to CD200R1, despite occurring at a distinct site from the CD200 interaction interface, may either block CD200 by steric hindrance as observed for the 23ME-00610 antibody (63) or induce a conformational change in CD200R1. This structural alteration, while not impeding the CD200/CD200R1 interaction, could potentially disrupt the intracellular signaling cascade typically initiated by this axis. Such a mechanism would provide a plausible explanation for the observed biological effects. This hypothesis aligns with the concept of allosteric modulation, where ligand binding at one site can influence protein function at a distal site.

Alternatively, similar to the synthetic peptide CD200AR-L, BOC1 could potentially bind to CD200 activation receptors (CD200ARs in mice or their human equivalent hCD200RLa) (41, 64). These activation receptors are characterized by a short cytoplasmic chain that interacts with adaptor proteins containing immunoreceptor tyrosine-based activation motifs (ITAM), which serve as anchor points for intracellular signaling. Recently, Olin and colleagues developed a CD200 homolog, CD200AR-L, derived from residues 32–44 of CD200 (corresponding to 42% of the CD200 binding site to CD200R1), which specifically targets the CD200AR complex (64, 65). They demonstrated that CD200AR-L binding to CD200AR upregulates the expression of the transmembrane signaling adaptor proteins DAP10 and DAP12 while downregulating CD200R1. Furthermore, they observed that human CD200AR-L activates immune upregulation by inducing a cytokine response on CD14^+^ cells and promoting dendritic cell differentiation (65). Notably, DAP10, predominantly expressed in CD8^+^ T cells and monocytes, is crucial for tumor control. A similar mechanism of action could be envisioned for BOC1; however, further studies are required to support the hypothesis of a possible interaction between BOC1 and CD200ARs.

The question of whether a bacterial protein needs to interact with CD200R1 is open. The more plausible hypothesis is to evade the host immune response; however, our current results do not agree with this assumption. This unknown protein of 201 amino acids is highly similar (99% and 67% sequence identity, respectively) to proteins of *B. gallinaceum* and *P. plebeius*, which is quite intriguing since *B. gallinaceum* is a bacterium found in chickens. Additionally, BOC5, the protein homologous to BOC1 (67% identity), presents a sequence highly matching (99.5%) with a protein of *P. plebeius*. Recent phylogenetic analysis has reclassified *B. gallinaceum* (and some other *Bacteroides* species) in the *Phocaeicola* genus (66). Furthermore, when looking at the phylogenetic tree, it is interesting to note that BOC1 is classed within a small clade of three proteins, which is close to a bigger clade clustering 34 homolog proteins of *P. plebeius* (**Supplementary Figure 4**). We may imagine that all these 37 proteins share similar functions. It is noteworthy that bacteria may grow on tumor cells and produce compounds (metabolites or proteins) that either inhibit or stimulate antitumor response. *Phocaeicola plebeius* is an intriguing bacterium since it colonizes prevalently the gut of Japanese people whose daily diet contains seaweeds (40). This bacterium contains various carbohydrate-active enzymes (CAZymes) (67) presumably coming through horizontal gene transfer from marine bacteria associated with the *Porphyra* spp. seaweed (68). Metabolites derived from seaweed fiber degradation by *P. plebeius* CAZymes were recently shown to reduce inflammation and colon cancer progression in the azoxymethane-dextran sulfate mice model (69). The same bacterium has also been described to improve muscle wasting in chronic kidney disease through gut microbiome modulation (70). We cannot definitively assess that BOC1 is a protein from *P. plebeius* or that *P. plebeius* is an “anti-inflammatory” bacterium, but we like to believe that some microorganisms can affect host response to disease through complex mechanisms involving multiple effectors. While more in-depth investigations, mainly *in-vivo* studies, are required to assess BOC1 potency (alone or in combination with other ICIs) and safety, these preliminary results confirm once more the gut microbiota as a key factor in shaping host immune functions.

## Data Availability

The raw data supporting the conclusions of this article will be made available by the authors, without undue reservation.
