# Supplementary figures and images for: The gut microbiota protein BOC1 exhibits immune checkpoint inhibitor-like activity by inhibiting myeloid-derived suppressor cell differentiation
Source: Front Immunol. 2025 Aug 19;16:1607543. doi: 10.3389/fimmu.2025.1607543 (PMC12401969; doi:10.3389/fimmu.2025.1607543)

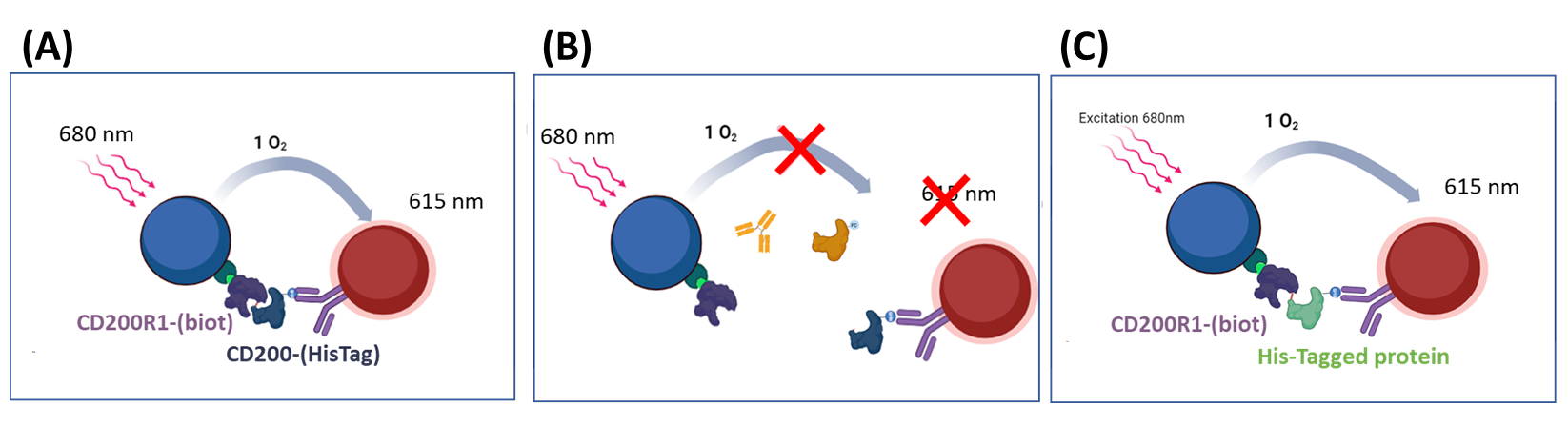

Supplement: Supplementary file 1 [file Image1.tif]

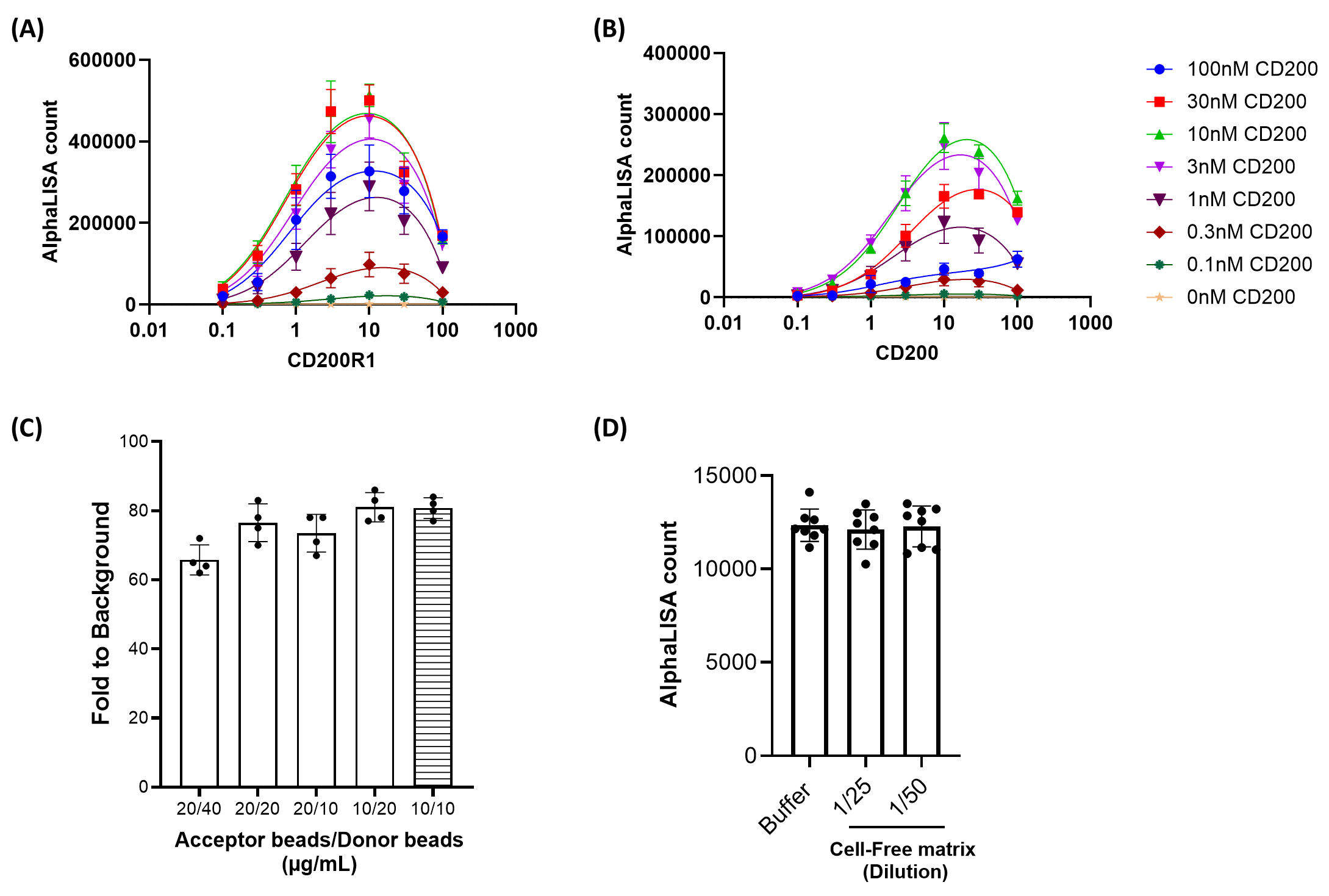

Supplement: Supplementary file 2 [file Image2.tif]

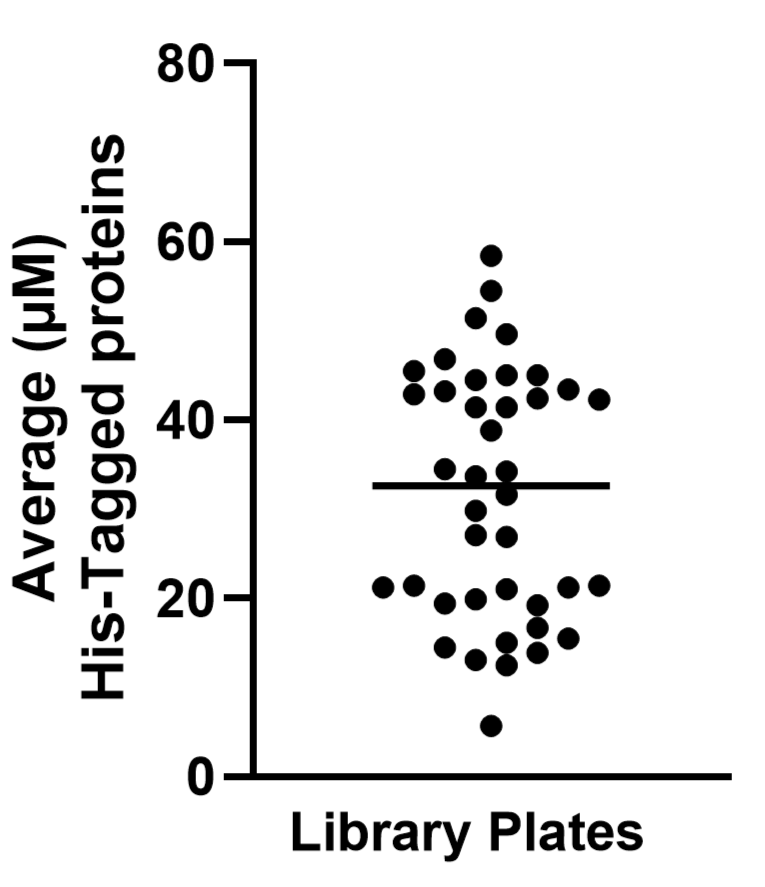

Supplement: Supplementary file 3 [file Image3.tif]

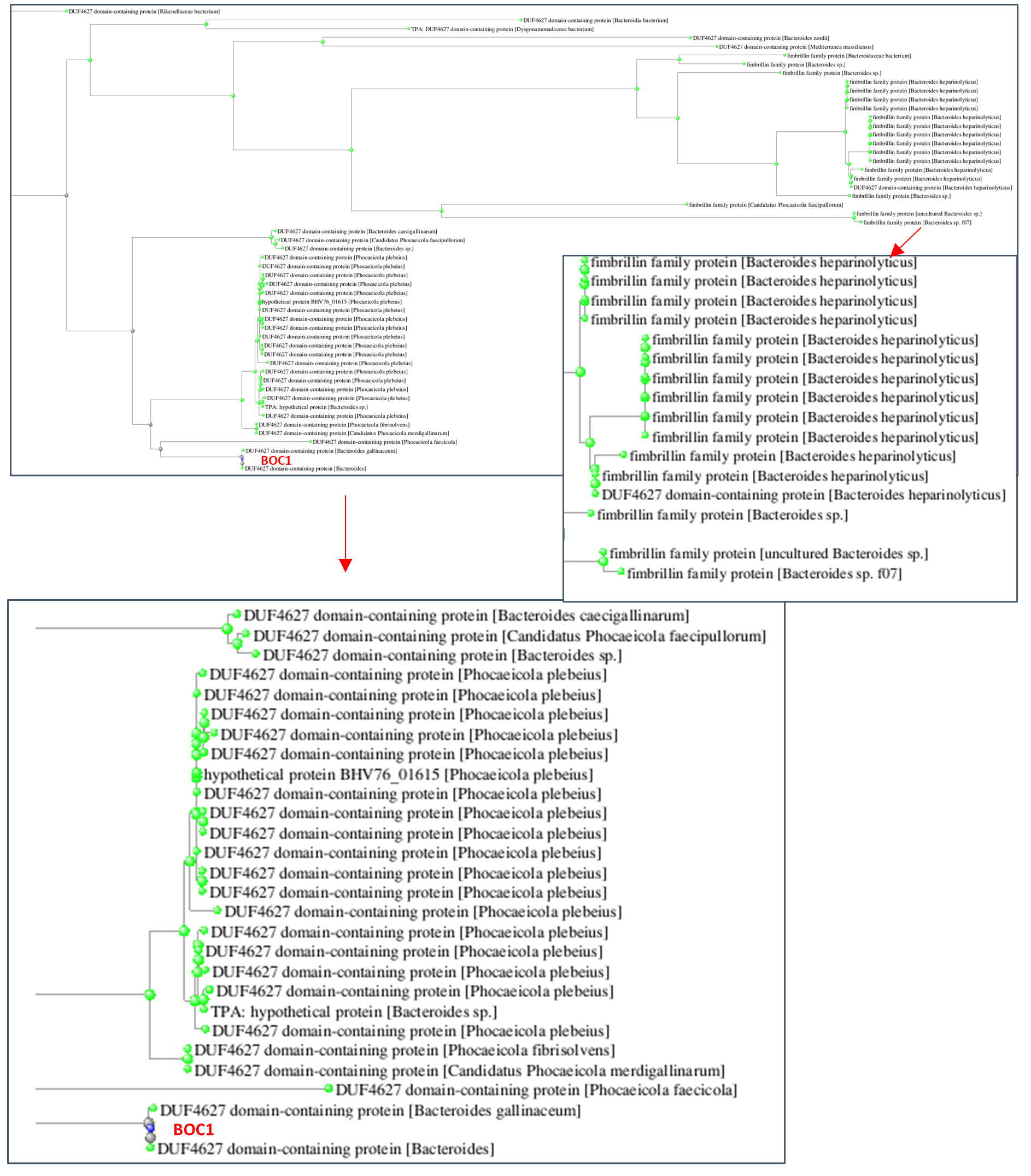

Supplement: Supplementary file 4 [file Image4.tif]

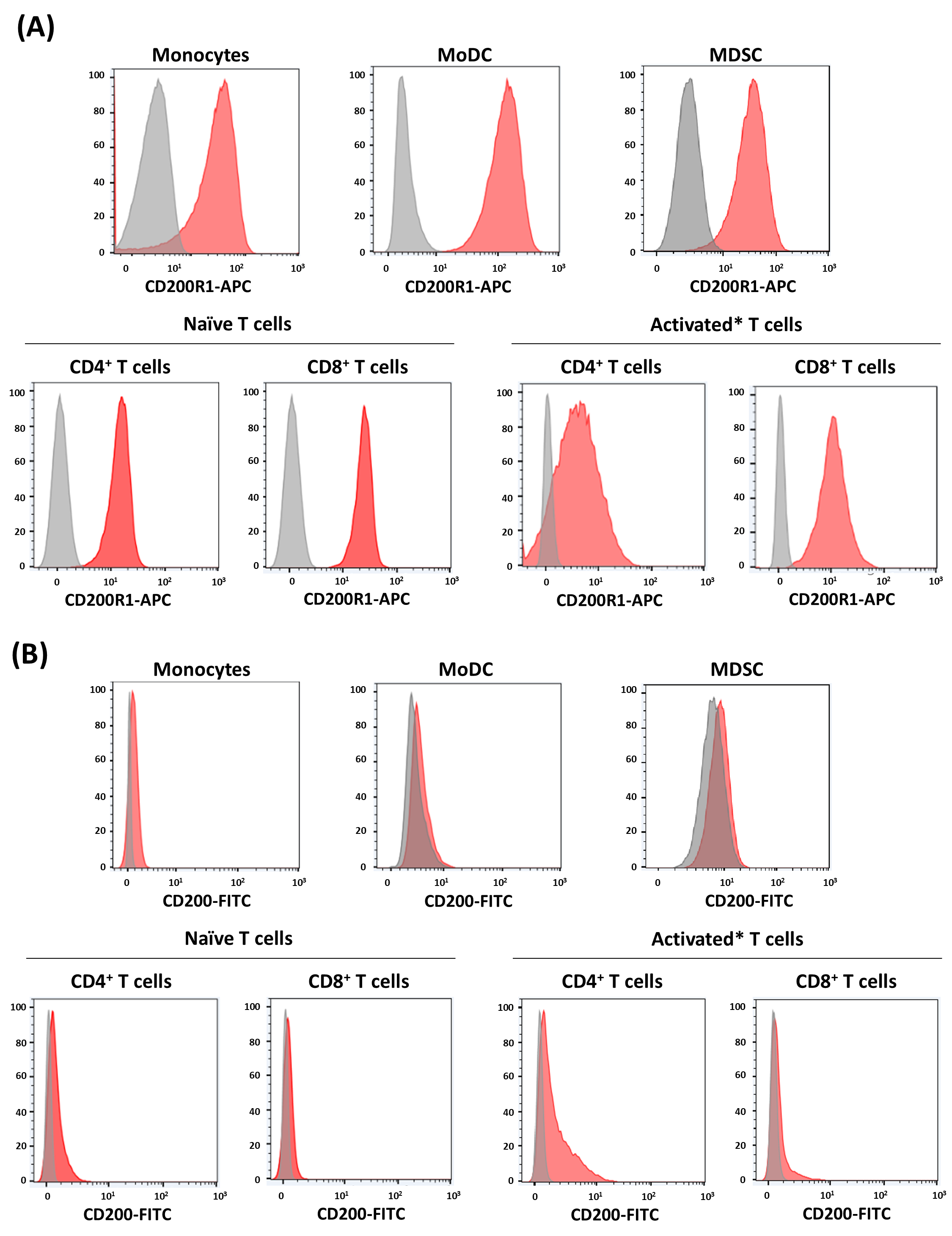

Supplement: Supplementary file 5 [file Image5.tif]

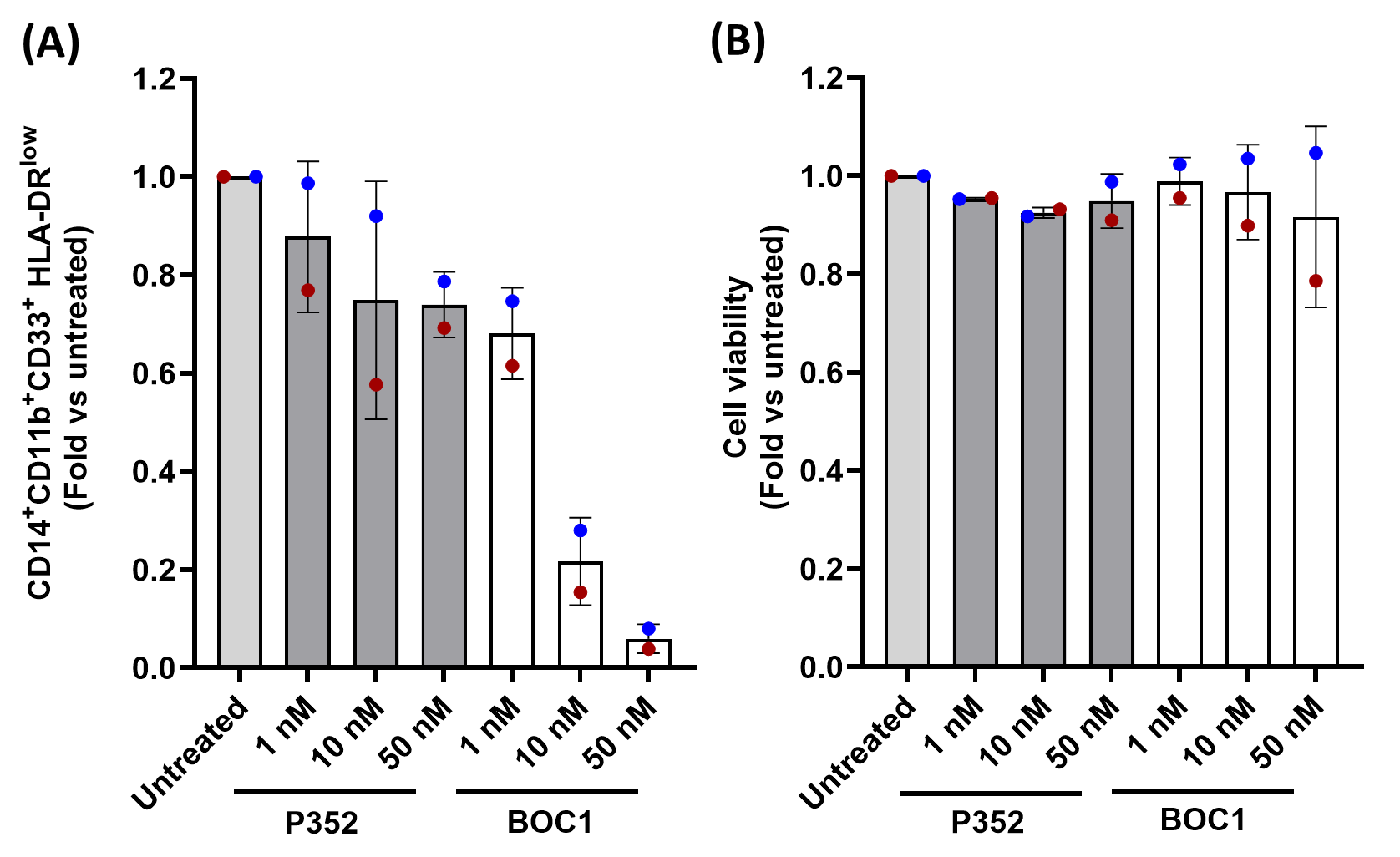

Supplement: Supplementary file 6 [file Image6.tif]
