# Supplementary material for: The gut microbiota protein BOC1 exhibits immune checkpoint inhibitor-like activity by inhibiting myeloid-derived suppressor cell differentiation
Source: Front Immunol. 2025 Aug 19;16:1607543. doi: 10.3389/fimmu.2025.1607543 (PMC12401969; doi:10.3389/fimmu.2025.1607543)
Supplement: Supplementary file 7 [file Table1.docx]

Supplementary information

The gut microbiota protein BOC1 exhibits immune checkpoint inhibitor-like activity by inhibiting myeloid-derived suppressor cell differentiation

Laureen Bardouillet^1^, Maria Lucia Orsini Delgado^1^, Caroline Matondo^1^, Francesco Strozzi^1^, Valentine Thomas^1^, Laurent Chene^1^, Antonietta Cultrone^1*^

^1^Drug Discovery Department, Enterome, Paris, France

*** Correspondence:**

Antonietta Cultrone

[antonellacultrone@gmail.com](mailto:antonellacultrone@gmail.com)

# CD200/CD200R1 AlphaLISA development & Screening setup

To identify new potential ligands of CD200R1 from a library of bacterial proteins issued from human gut microbiota we developed an AlphaLISA CD200/CD200R1 (**Suppl. Fig.1**). CD200-His, CD200-biot, CD200R1-biot and CD200R1-His proteins were from Acrobiosystem. Anti-6His Acceptor Beads and streptavidin Donor beads were from Revvity. All reagents and proteins dilutions were prepared in ImmunoAssay buffer (Revvity).

We first identified the optimal protein concentrations to be used in the screening through a cross titration (from 0.3 nM to 300 nM) of each protein. As CD200 and CD200R1 are both available with C-terminal His-Tag or biotinylation, both combinations were tested for cross-titration (CD200-(biot)/CD200R1-His-Tag or CD200R1-(biot)/CD200-His) (**Suppl. Fig. 2A-B**). The best signals were obtained with the couple CD200R1-(biot)/CD200-His (**Suppl.** **Fig. 2A**). 0.3 nM CD200R1-(biot) and 3 nM CD200-His, generating a signal corresponding to 30% of the maximum signal, were chosen as the best concentrations for the screening. Under these conditions, no hook effect was reached, and a sufficient window to further increase the signal was available.

Secondly, we determined the optimal beads concentration (using a fixed protein concentrations established at 0.3 nM CD200R1-(biot) and 3 nM CD200-His). Three concentrations of both acceptor anti-6His beads and donor streptavidin beads were tested (10 - 20 and 40 µg/mL). The signal got from the interaction between the beads and the protein was compared with the background noise generated by the beads incubated with the immunoassay buffer alone (**Suppl. Fig. 2C**). The optimal concentrations of beads used for the screening and hit characterization was 10 µg/mL for both acceptor and donor beads. Finally, we verified that the matrix of the cell free kit did not cause any interference with the AlphaLISA assay. To this aim, two mix dilutions (1:25 and 1:50 in PBS) were used to dilute the 0.3 nM CD200R1-biot and the 3 nM CD200-His, and the AlphaLISA signal was compared to the signal generated by the same proteins diluted in the Immuno assay buffer (**Suppl. Fig. 2D**). No interference of the cell free kit was observed thus validating the screening protocol.

# Supplementary figures’ legends

**Suppl. Figure 1**. **CD200/CD200R1 AlphaLISA setup layout (A)** Biotinylated-CD200R1 (Violet) bound to streptavidin-coupled beads (Blue bead) interacts with His-tagged CD200 (Dark Blue) bound to anti-6His coupled beads (Red bead). Following excitation at 680 nm the interaction between the two proteins generates an AlphaLISA signal measurable at 615 nm. (**B**) Competitive AlphaLISA in which a competitor (a protein or an antibody, indicated in Yellow) can interact with one of the two partners described in the first panel (A) and stop the emission at 615 nm. (**C**) Screening condition allowing the identification (light emission at 615 nm) of those His-tagged proteins (indicated in Green and bound to anti-His coupled beads) that are able to interact with the biotinylated-CD200R1 (bound to streptavidin-coupled beads).

**Suppl. Figure 2. CD200/CD200R1 AlphaLISA setup** (A) Cross-titration CD200-His/CD200R1-(biot). (B) Cross-titration CD200R1-His/CD200-(biot). Proteins were tested between 0.1 – 100 nM. (C) AlphaLisa beads optimization: ratio (µg/mL) anti-6His conjugated acceptor beads / streptavidin-coated donor beads. Ratio 10:10 (striped bar) was selected for screening. (D) Effect of Cell-free synthesis matrix on AlphaLISA signal and comparison with signal (background) generated by AlphaLISA buffer. Data (A-B-D) are expressed as AlphaLISA signal and represent the mean of 2 independent experiments (each experiment including 4 replicates/point). Error bars represent the SD. Data (C) are expressed as Fold to background and are shown as mean ± SD of quadruplicate measurements of a representative of two independent experiments.

**Suppl. Figure 3**. **HTRF-6His quantification of Cell-free produced proteins.** Average (µM) of His-tagged proteins in each plate (384 wells) of the library. Each dot represents one library plate

**Suppl. Figure 4**. Distance tree of protein sequences matching with BOC1 obtained by blast pairwise alignment.

**Suppl. Figure 5.** CD200R1 (A) and CD200 (B) expression on different cell types. Cells were incubated with fluorescently labelled antibodies directed against CD200 and CD200R1, and MFI determined by flow cytometry. Representative results of a single donor. *Activated with antiCD3/CD28 for 4 days.

**Suppl. Figure 6.** Dose effect (1 – 10 – 50 nM) of BOC1 (white bar) or P352 (dark grey bar) on the inhibition of monocyte differentiation into MDSC (A) and on cell viability (B). Representative results of a single experiment using monocytes from two blood donors. Data are expressed as Fold versus Untreated cells (mean ± SD).
